# Analysis of Dual Class I Histone Deacetylase and Lysine Demethylase Inhibitor Domatinostat (4SC-202) on Growth and Cellular and Genomic Landscape of Atypical Teratoid/Rhabdoid

**DOI:** 10.3390/cancers12030756

**Published:** 2020-03-23

**Authors:** Mariah M. Hoffman, Jessica S. Zylla, Somshuvra Bhattacharya, Kristin Calar, Timothy W. Hartman, Ratan D. Bhardwaj, W. Keith Miskimins, Pilar de la Puente, Etienne Z. Gnimpieba, Shanta M. Messerli

**Affiliations:** 1 Department of Biomedical Engineering, University of South Dakota, Sioux Falls, SD 57107, USA; Mariah.Hoffman@coyotes.usd.edu (M.M.H.); Etienne.Gnimpieba@usd.edu (E.Z.G.); 2 Department of Biomedical Engineering, University of South Dakota, BioSNTR, Sioux Falls, SD 57107, USA; 3 Cancer Biology & Immunotherapies, Sanford Research, Sioux Falls, SD 57104, USAPilar.Puente@sanfordhealth.org (P.P.); 4 Department of Surgery, University of South Dakota Sanford School of Medicine, Sioux Falls, SD 57105, USA; 5 Department of Chemistry and Biochemistry, South Dakota State University, Brookings, SD 57006, USA

**Keywords:** ATRT, 4SC-202, Domatinostat, cancer stem cell, scaffold, single-cell RNA-sequencing, systems biology

## Abstract

Central nervous system atypical teratoid/rhabdoid tumors (ATRTs) are rare and aggressive tumors with a very poor prognosis. Current treatments for ATRT include resection of the tumor, followed by systemic chemotherapy and radiation therapy, which have toxic side effects for young children. Gene expression analyses of human ATRTs and normal brain samples indicate that ATRTs have aberrant expression of epigenetic markers including class I histone deacetylases (HDAC’s) and lysine demethylase (LSD1). Here, we investigate the effect of a small molecule epigenetic modulator known as Domatinostat (4SC-202), which inhibits both class I HDAC’s and Lysine Demethylase (LSD1), on ATRT cell survival and single cell heterogeneity. Our findings suggest that 4SC-202 is both cytotoxic and cytostatic to ATRT in 2D and 3D scaffold cell culture models and may target cancer stem cells. Single-cell RNA sequencing data from ATRT-06 spheroids treated with 4SC-202 have a reduced population of cells overexpressing stem cell-related genes, including *SOX2*. Flow cytometry and immunofluorescence on 3D ATRT-06 scaffold models support these results suggesting that 4SC-202 reduces expression of cancer stem cell markers SOX2, CD133, and FOXM1. Drug-induced changes to the systems biology landscape are also explored by multi-omics enrichment analyses. In summary, our data indicate that 4SC-202 has both cytotoxic and cytostatic effects on ATRT, targets specific cell sub-populations, including those with cancer stem-like features, and is an important potential cancer therapeutic to be investigated in vivo.

## 1. Introduction

Atypical teratoid rhabdoid tumor (ATRT) is a type of rare and aggressive central nervous system tumor with poor prognosis; the median survival is only 6–17 months [1,2,3,4,5,6,7]. A malignant embryonal tumor of the central nervous system (CNS), ATRT is composed of primarily rhabdoid cells that may or may not have fields resembling classical primitive neuroectodermal tumor [8]. Tumors can occur in any location in the CNS, including the spinal cord [9], and a majority of cases are characterized by the deletion or mutation of the INI1 gene [10]. ATRTs are a rare disease, comprising only 1–2% of all pediatric CNS tumors – but they make up between 10–20% of malignant brain tumors for patients under the age of 3 [3,10,11].

Surgery and radiation alleviate imminent neurological problems but are accompanied by exceedingly morbid long-term side effects. Aggressive multiple chemotherapy approaches to substitute radiation have been attempted, yet overall survival is dismal, particularly for patients under three years of age [5,6,7]. This aggressive tumor remains a challenge in the field of pediatric neuro-oncology and newer therapeutic approaches are desperately needed to increase survival for very young patients.

The drug 4SC-202 is a recently developed histone deacetylase inhibitor (HDACi) that has not been fully characterized in terms of its various applications or mechanisms of action. 4SC-202 is an orally available benzamide-type HDACi that specifically targets class I HDACs—HDAC1, HDAC2, HDAC3, and HDAC8—and the histone demethylase LSD1 (4SC company data: B.P.S. Bioscience Assay Report, Reaction Biology Corporation Assay Results) and is more potent and specific than currently available HDAC inhibitors [12,13]. In multiple cancer cell lines and preclinical models, 4SC-202 has exerted anti-tumor activities [13,14,15]. We have previously published that 4SC-202 significantly reduces the viability of medulloblastoma in culture [16]. Pre-clinical studies performed in hepatocellular carcinoma, medulloblastoma, urothelial carcinoma, and colon cancer indicate that 4SC-202 inhibits cell proliferation, induces apoptosis, inhibits mitosis, and is efficacious in vivo, causing a reduction of tumor growth in a number of different mouse xenograft studies including Hedgehog driven basal cell carcinoma (BCC) and a colorectal cancer murine model [12,13,15,17,18]. A first-in-humans clinical study showed that the drug is safe and has potential anti-cancer benefits in hematological cancers [19].

While preclinical studies have demonstrated the efficacy of 4SC-202 in some models, the mechanism by which 4SC-202 exerts anti-cancer effects stills needs further characterization. Prior studies indicate 4SC-202 may target cancer stem cells [20] and other studies have demonstrated that 4SC-202 blocks hedgehog (HH)/Gli signaling in a human medulloblastoma model [12,16]. Hedgehog signaling has been shown to play a role in the maintenance of stem cells [21]. Cancer stem cells (CSCs) comprise a small percentage of tumors (0.01–1%) within a heterogeneous tumor mass [22,23,24], are able to self-renew, and have been implicated in cell migration and metastasis as well as treatment resistance [25]. Since 4SC-202 blocks hedgehog signaling, which has been implicated in the maintenance and survival of stem cells [26,27,28], we hypothesize that 4SC-202 may influence the stemness of the ATRT cancer stem cell population and thereby alter the growth and survival of ATRT.

## 2. Results

### 2.1. ATRTs Have Aberrant Expression of 4SC-202 Targets

It has been previously demonstrated that ATRTs are good candidates for HDACi treatment due to epigenetic dysregulation [29]. These conclusions correspond well with the results from a reanalysis of a previously published custom NanoString nCounter gene expression panel comparing frozen ATRT tumors (*n* = 17) to frozen normal pediatric neuronal samples (*n* = 7), where the top differentially expressed genes (DEGs) include epigenetic modulators involved in acetyl and methyl regulation (Figure S1). Since 4SC-202 not only functions as a Class I histone deacetylase inhibitor (HDACi) but also influences methylation by inhibiting lysine demethylase (LSD1), the specific targets of 4SC-202 were evaluated to determine if these genes are upregulated in ATRT in order to determine the suitability of 4SC-202 for ATRT treatment.

**Figure 1:**
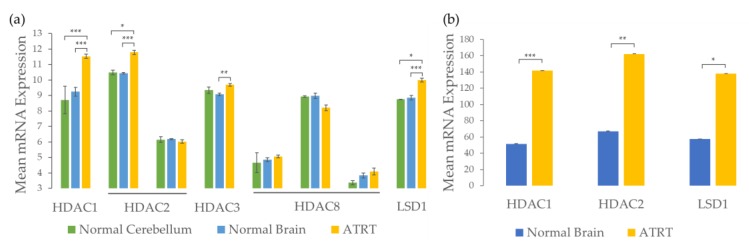
Gene expression analysis indicates epigenetic targets such as class I HDAC’s (HDAC 1, 2, 3, 8) and LSD1 are upregulated in ATRT tissue relative to normal brain tissue. (**a**) Analysis of GSE35493 [29] microarray data demonstrates significant upregulation of most Class I HDAC’s and Lysine Demethylase (LSD1) in ATRT tumors compared to normal pediatric brain tissue samples (*n* = 7 normal brain tissue samples, *n* = 17 ATRT tissue samples). (**b**) Reanalysis of raw NanoString data [30] confirms upregulation of *HDAC* 1, 2, and *LSD1* in ATRT tumor tissue compared to age-matched normal brain tissue (*n* = 7 normal brain tissue samples, *n* = 17 ATRT tissue samples). Error bars represent the standard error of the mean. P values were adjusted using the Benjamini and Hochberg procedure; *p* < 0.001 (***), *p* < 0.01 (**), *p* < 0.1 (*).

Further analysis of previously published microarray and NanoString gene expression datasets from human ATRT tissue samples and normal age-matched brain samples [30] suggest that Class I HDACs, *HDAC1, HDAC2, HDAC3*, and epigenetic modifier *LSD1* are significantly overexpressed in ATRT when compared to normal brain samples (Figure 1). *HDAC1* and *LSD1* are significantly overexpressed in both datasets. *HDAC2* is overexpressed in the NanoString dataset for the fresh frozen tissue, but only overexpressed in one of the two probesets for the microarray. The probeset that is overexpressed (201833_at) includes more transcripts than the probeset that is not (242141_at). *HDAC3* is significantly overexpressed compared to normal brain in the microarray dataset and has higher mean expression in ATRT than normal cerebellum samples, but the difference is not significant, possibly due to small sample size (*n* = 2 normal cerebellum samples). Differences in expression of *HDAC8* are not significant for any of the probesets in the microarray dataset. These results are consistent with the original analysis of the microarray data that found that ATRTs were characterized by dysregulation of epigenetic markers [29]. Because of this epigenetic dysregulation involving overexpression of *HDAC1*, *HDAC2*, *HDAC3*, and *LSD1*, we anticipate that the 4SC-202, which specifically targets Class I HDACs (HDAC1, HDAC2, HDAC3, HDAC8) and LSD1, may selectively affect ATRT tumor cells.

### 2.2. 4SC-202 Is Cytotoxic and Cytostatic to ATRT in Two- and Three- Dimensional Cell Culture

**Figure 2:**
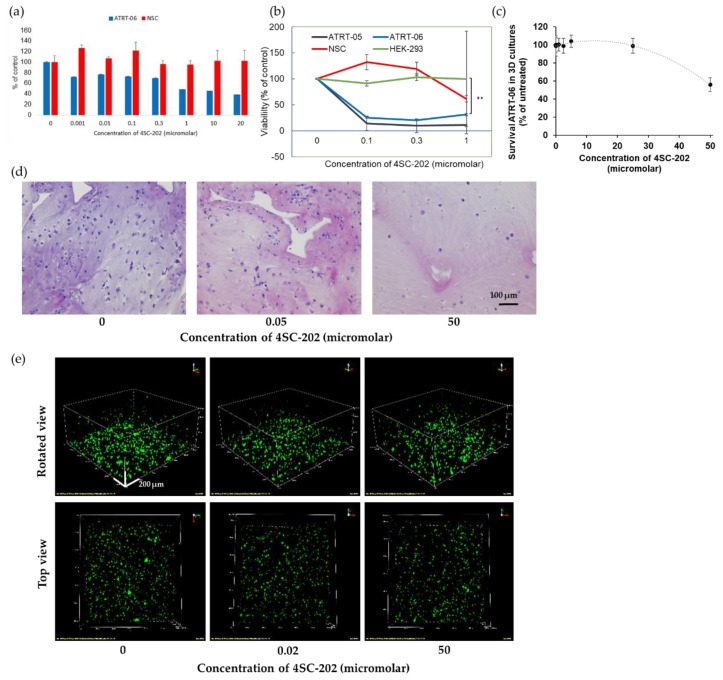
4SC-202 is significantly cytotoxic and cytostatic to ATRT in 2D and 3D cell culture models. (**a**) Sytox Green proliferation assays indicate that 4SC-202 significantly reduces live cells in ATRT-06 at concentrations ranging from 0–20 µM as compared to a control neural stem cell (NSC) line. (**b**) Luminescence viability assays (Promega) indicate 4SC-202 significantly reduces viability in 2 separate ATRT cells lines-ATRT-06 and ATRT-05 at concentrations ranging from 0–1 micromolar but does not affect viability of non-cancerous cell lines NSC and HEK-293. A two-tail paired t-test indicates that at 1 µM 4SC-202 treatment, there is a significant difference in viability between ATRT-06 as compared to HEK-293, *p* < 0.001 (**), and other significant differences between ATRT cell lines as compared to controls noted in text. (**c**) 4SC-202 reduces survival of ATRT-06 in 3-dimensional scaffold model. (**d**) H&E confirm a dose-dependent reduction in proliferation, at concentrations ranging from 0–50 µM with the fewest cells visible following 50 µM 4SC-202. Scale bar = 100 μm (**e**) Representative confocal microscopy images of rotated and top views of ATRT-06 cell line (DiO, green) in 3D matrices on day 3 at concentrations ranging from 0–50 µM. Scale bar = 200 μm.

In two-dimensional cell culture, 4SC-202 was significantly cytotoxic to two ATRT cell lines, ATRT-06 and ATRT-05, following 72 h. of nanomolar- to micromolar-scale drug exposure but did not affect the viability of non-cancer cell lines—neural stem cells (NSC) and human embryonic kidney (HEK-293) cells—in several separate experiments. Significant differences in viability were observed between ATRT-06 as compared to NSC (*p* < 0.05), and between ATRT-06 and ATRT-05 compared to HEK-293 (*p* < 0.001), at 1 µM 4SC-202 treatment, according to paired two-tailed t-tests (Figure 2a,b). Recent studies examining the molecular subtyping of ATRT tumors indicate that ATRT-05 is most closely correlated with Group 1 ATRT (neurogenic, ATRT-SHH) and ATRT-06 with Group 2 ATRT (mesenchymal, ATRT-MYC) [31,32]. Additionally, in a spheroid model, treatment with 56 nM 4SC-202 significantly decreased spheroid growth when compared to a vehicle treatment with 0.02% DMSO (Figure S2). A higher dose of Domatinostat (4SC-202) reduced ATRT-06 cell growth within 3D scaffolds. As illustrated in Figure 2c, ATRT-06 cells grown within the 3D scaffold niche exhibited lower survivability than when treated with 50 μM 4SC-202. Flow cytometry experiments demonstrated that almost50 % of ATRT-06 cells were dead when exposed to 50 μM 4SC-202 within the scaffolds. These findings were corroborated by H&E staining (Figure 2d) of cell-laden scaffold section slices, where the number of eosin-stained ATRT-06 cell nuclei was observed to reduce with an increase in 4SC-202 concentration. Additionally, these findings were also confirmed by confocal imaging of 3D scaffolds embedded with ATRT-06 cells (DiO, green). An increase in 4SC-202 concentration resulted in loss of ATRT-06 cells as demonstrated by H&E staining (Figure 2d) and DiO staining of the scaffold sections (Figure 2e). Results from an image analysis of the confocal images with Fiji indicate that 50 µM 4SC-202 changes the cells’ spatiotemporal distribution (clustering) in the segmented 3D portion of the model relative to no treatment (Videos S1–S4).

These changes in the cells’ spatiotemporal distribution in the scaffolds were semi-quantitatively characterized by analyzing the frequency of the volumes of segmented cells (Figure S3). Changes in the volume density indicate that there is a change in the spatiotemporal distribution pattern with an increase in the relative number of clustered cells and size of the cell clusters in the controls as compared to the sample treated with 0.2 µM 4SC-202. In contrast, for the 50 µM 4SC-202-treated sample, the relative number of non-clustered cells increased (Figure S3).

These results in both a spheroid and 3D scaffold model of ATRT demonstrate that 4SC-202 selectively decreases the viability of ATRT cells.

### 2.3. 4SC-202 Decreases the Population of Cells Overexpressing Stem Cell Markers

**Figure 3:**
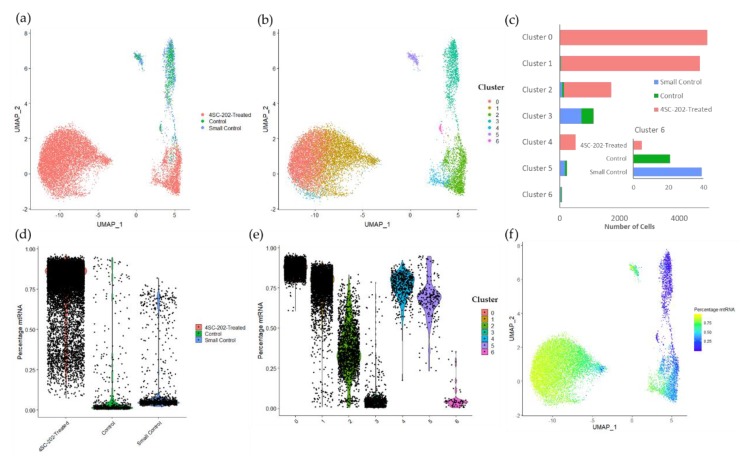
Single cell RNA-sequencing (scRNA-seq) data clustering corresponds to differences in mtDNA expression levels and treatment group. (**a**) Depiction of Uniform Manifold Approximation and Projection (UMAP) dimensional reduction of integrated early untreated spheroid, vehicle-treated spheroid, and 4SC-202-treated spheroid scRNA-seq transcriptomic data. (**b**) UMAP plot of graph-based clustering of integrated scRNA-seq datasets. Clustering algorithm optimizes to 7 clusters. (**c**) Number of cells per cluster by sample group. Inset: Cluster 6 by sample group. (**d**) Violin plot of the percentage of raw reads that are mapped to mtRNA across experimental conditions. (**e**) Violin plot of the percentage of raw reads mapped to mtRNA across clusters. (**f**) UMAP plot of integrated spheroids colored by the percentage mtRNA.

Because there is preliminary evidence that 4SC-202 may affect cancer cell stemness or stem cell-related pathways [12,20], a population-based analysis was conducted to characterize how 4SC-202 induced changes in specific cell populations in ATRT. Single-cell RNA-sequencing was performed on early smaller untreated spheroids (7 days after plating, small control), and larger spheroids treated with either 0.02% DMSO (large control) or 56 nM 4SC-202 (9 days after plating) (Figure 3a). Because the user does not select the number or occupancy of clusters in the graph-based clustering method used, clustering was used as a tool to identify populations in the single cell dataset. Cells cluster principally according to (1) experimental condition and (2) health of the cells, though these factors alone are insufficient to account for cluster 6 (Figure 3b). The small control and the large control spheroids are well integrated and group together both in the Uniform Manifold Approximation and Projection (UMAP) representation and in the clustering. Cells from the drug-treated spheroid are generally clustered separately with only a small percentage of cells overlapping either in the UMAP representation or the clusters (Figure 3b,c). These results suggest that the difference between the small and large control spheroids are significantly smaller than the differences between the drug and control spheroids, as would be expected since HDACi are known to change gene expression patterns [33].

In addition to experimental conditions, cells also appear to be clustered based on the health of the cell. Clusters 0, 1, 4, and 5 all have a high percentage of their genes mapped to mitochondrial genes (high mtRNA) whereas clusters 2, 3, and 6 have a lower percentage of mitochondrial genes (low mtRNA) (Figure 3e,f). High mtRNA content has been correlated with broken cells where cytoplasmic content has been lost but mitochondrial content has been better retained [34]. Of the low mtRNA content cell clusters, clusters 3 and 6 are predominantly control, whereas cluster 2 is principally 4SC-202-treated (Figure 3). 4SC-202-treated cells have higher mtRNA expression than the control cells. 74.9% and 76.7% of small and large control spheroid cells have a percent mtRNA under 10 %, respectively, whereas only 0.094 % of 4SC-202-treated cells have a percent mtRNA under 10% (Figure 3d). While for the downstream analysis clusters 2, 3, and 6 are considered “healthy”, the drug-treated cluster 2 may be less healthy than the clusters principally populated by control tumor cells (Figure 3e).

Gene expression of class I HDACs are altered in 4SC-202-treated cluster 2 relative to control clusters 3 and 6 (Figure S4a). *HDAC1* and *HDAC2* are significantly underexpressed in the 4SC-202-treated cluster relative to control. *HDAC3* is overexpressed in the 4SC-202-treated cluster relative to the control clusters and *KDM1A* and *HDAC8* appear to be overexpressed in cluster 6 relative to the other clusters, though not significantly. This lack of significance for cluster 6 relative to the other clusters may be due to the small number of cells in cluster 6 (*n* = 65) relative to clusters 3 (*n* = 1142) and cluster 2 (*n* = 1718). Because *HDAC1* and *HDAC2* are overexpressed in ATRT relative to normal brain, 4SC-202 may alter pathways related to dysregulation of these genes. The change in the expression of genes critical for the replication of ATRT tumorigenesis in vivo were also tested (Figure S4b) [35]. While *TP53* shows no significant difference across clusters 2, 3, and 6, *SMARCB1* is underexpressed in 4SC-202-treated cluster 2 relative to cluster 6. This result suggests that 4SC-202 does not rescue the expression of genes critical to the formation and development of ATRT.

While most of the clustering illustrated in Figure 3 can be attributed to differences between treatment group and mtRNA expression levels, these factors alone are unable to explain the difference between clusters 3 and 6, which are both principally control clusters and have low percent mtRNA. Cluster 6 accounts for 4.53% of the “healthy” control cells, but only 0.31% of the “healthy” drug-treated cells. Most “healthy” 4SC-202-treated cells are localized in cluster 2, and there is no equivalent of cluster 6 for the drug-treated data. 

**Figure 4:**
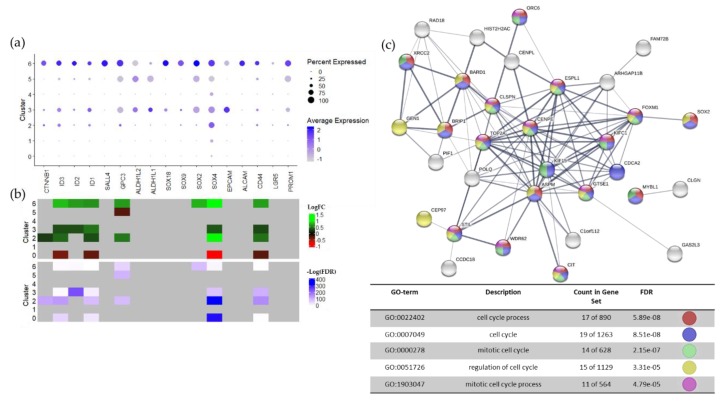
Cluster 6 overexpresses stem cell markers and cell cycle biological processes. (**a**) Dot plot visualization of the average expression and percentage of cells expressing stem cell markers or stem cell-related genes across clusters generated in Seurat. Color of the dot indicates the average expression of the feature and the size indicates what percentage of the cells express the feature. (**b**) Log_2_ fold change (LFC, top heatmap) and significance of stem cell marker expression (bottom heatmap) across clusters. Significance is calculated using the Wilcox Rank Sum test in Seurat [36,37] (*p* < 0.01, |LFC| > 0.25). Green indicates positive log fold expression changes and red indicates negative log fold expression changes. Non-significant changes are grey. Significance is shown as the negative log of the FDR. No significance is grey, low significance is white, and high significance is blue. Heatmaps were generated with the ComplexHeatmap package in R [38]. (**c**) STRING protein–protein interaction network [39] and GO biological processes enrichment for the 50 stably differentially expressed genes with the lowest FDR. Only the five GO terms with the lowest FDR are shown. Nodes are colored according to involvement in enriched biological processes.

Cluster 6 overexpresses multiple stem cell markers, including *SOX2*, and stem cell-related genes relative to all other clusters and relative to low mtRNA control cluster 3 (Figure 4a,b; Figure S5). The average expression and percent of the cells expressing these stem cell-related genes are consistently higher in cluster 6 than in the other clusters. The log fold changes for these stem cell markers also appear to be higher for cluster 6 than for the other “healthy“ clusters, though the significance is lower. This discrepancy may be partially accounted for by the small size of cluster 6 (*n* = 65) compared to the other clusters (Cluster 2, *n* = 1718; Cluster 3, *n* = 1142) (Figure 3c). The overexpression of stem cell-related genes in cluster 6 is further supported by the overexpression of *ID2*, *ID3*, *GPC3*, *SOX2, SOX4*, and *CD44* in cluster 6 relative to the control cluster 3, which also has low percent mtRNA (Figure S5A). Overexpression of stem cell-related genes in normal cluster 6 relative to normal cluster 3 is supported by a larger panel of genes tested for local differential expression in Loupe Cell Browser and by ANOVA followed by a post-hoc Tukey’s test (Figure S5D–G). Globally, control ATRT samples also exhibit higher expression of stem cell-related genes relative to 4SC-202-treated samples, though this difference may be in part due to the difference in the relative sizes of the “healthy” and “unhealthy” populations (Figure S5B,C).

While cluster 6 appears to overexpress stem cell markers and related genes, the genes that are most overexpressed in this cluster (lowest FDR) are genes related to mitosis and cell cycling (Figure 4c). There was substantial overlap between the list of differentially expressed genes (DEGs) resulting from a comparison of cluster 6 to all clusters, and cluster 6 to control cluster 3, particularly for those genes with the highest significance (Figure S6). The protein–protein network for the consensus differentially expressed gene list built-in STRING [39] shows high levels of connectivity between these genes, though only one strong connection with SOX2 through FOXM1 (Figure 4c). The DEG are uniformly overexpressed and a significantly high percentage are related to mitosis and cell cycling gene ontology terms. This bias towards cell-cycle related genes as the top differentially expressed genes rather than stem cell markers may be related to the tendency of single cell RNA-sequencing results to emphasize highly expressed genes due to gene dropout events [40]. This also may account for the uniformly low expression of common stem cell markers such as *PROM1*.

**Figure 5:**
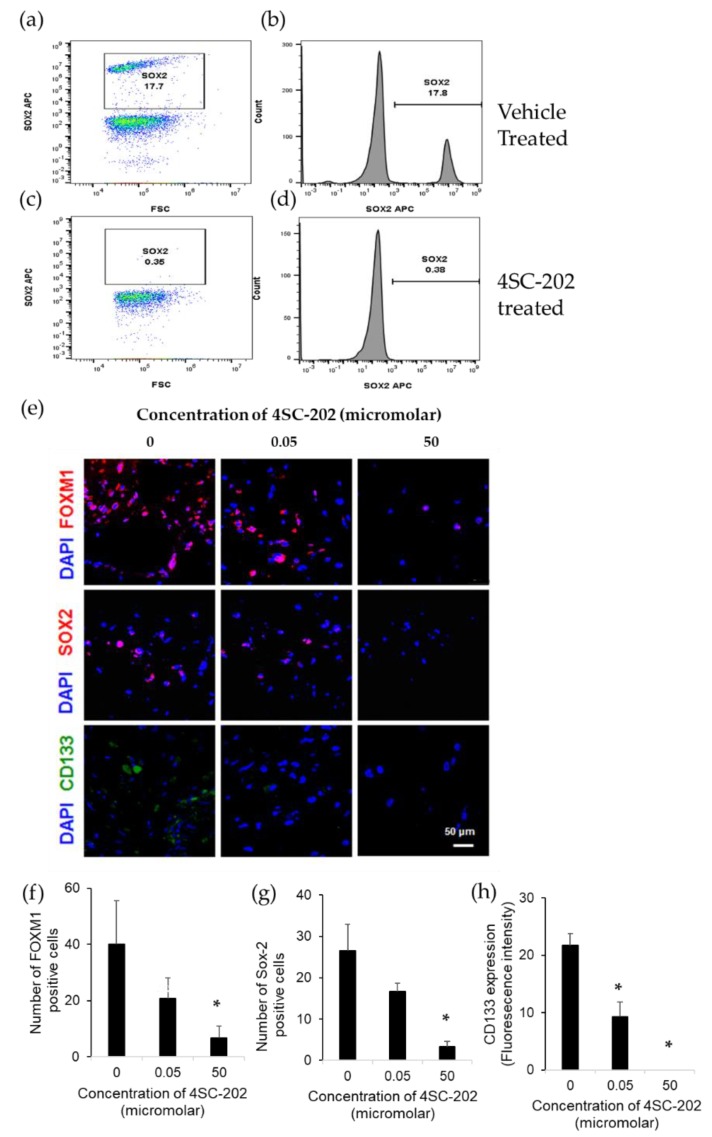
4SC-202 reduces a cancer stem-like population of cells. (**a**) A SOX2 positive population of cells in vehicle-treated ATRT-06 spheroids, indicated by a small peak (**b**), nearly disappears 72 h following 4SC-202 treatment (**c**,**d**). (**e**) Representative fluorescent images exhibiting reduced number of cells expressing FOXM1, SOX2, and CD133 by ATRT-06 cells grown in 3D scaffolds after treatment with 4SC-202 at μM concentrations. Scale bar = 50 μm. Stem cell marker expression of ATRT-06 cells growing within 3D scaffolds indicates a significant reduction in the number of FOXM1 positive cells (**f**), SOX2 positive cells (**g**), and CD133 expression (fluorescence intensity) (**h**), (*) *p* < 0.05.

4SC-202 was significantly cytotoxic to several different ATRT cell lines but did not affect viability of non-cancerous cell lines such as neural stem cells (Figure 2a,b). Flow cytometry experiments in ATRT spheroids indicate that a SOX2 positive population of cells (Figure 5a,b) is eliminated following 72 hours of 4SC-202 treatment (Figure 5c,d). Additionally, we evaluated stem cell marker expression after 4SC-202 treatment in 3D scaffolds. We found a dose-dependent reduction of FOXM1 and SOX2 expressing cells at µM range concentration of 4SC-202 and a complete CD133 expression reduction with 4SC-202 treatment (Figure 5e). Quantification of stem cell markers indicated a significant reduction of FOXM1 and SOX2 expressing cells (Figure 5f and Figure 5g, respectively) and complete elimination of CD133 expression with 50 µM 4SC-202 treatment after 3 days (Figure 5h).

Based on the significant decrease in the population of cells expressing stem cell markers after 4SC-202 treatment relative to the total population of surviving cells, 4SC-202 appears to reduce cell populations with high stem cell marker expression.

### 2.4. 4SC-202 Modulates Systems Biology Landscape in ATRT for Tumor Cell Population

Our understanding of the 4SC-202 mechanism of action toward the ATRT cell population with low stemness depends on its multi-omics systems biology landscape (transcription factors, gene, miRNA, protein, biological processes). The changes in the viability of the ATRT models cannot be explained exclusively by a change in a stem cell population since the population with high stemness characteristics is a minority of the total cells [41,42]. Consequently, it seems likely that cells with low stemness are also affected by 4SC-202. To identify potentially perturbed biological processes, the “healthy” control cluster (cluster 3) was compared to the “healthy” 4SC-202-treated cluster (cluster 2) from the single cell dataset. DEGs were considered those with an adjusted *p* value < 0.05. Because gene drop-out events are prevalent in single cell RNA sequencing datasets, DEGs were used as the basis to build networks across multiple systems biology levels including tissue-specific protein–protein interaction, gene-miRNA interaction, gene-TF interaction, and gene co-expression networks in NetworkAnalyst [43]. Over-representation analysis was run on the DEGs and each of these networks and the results compared systematically to identify consistent terms (FDR < 0.1). Those terms that were most consistent were grouped with other terms into manually curated term families that summarize families of potentially perturbed biological processes. While each individual enrichment test contributes partially to an understanding of the changes in the systems biology landscape, consistent and cumulative changes across each network increase the strength of the characterization of changes to the systems biology landscape. Detailed enrichment results for each systems biology level are available in the Supplemental Information (Figures S7–S11).

**Figure 6:**
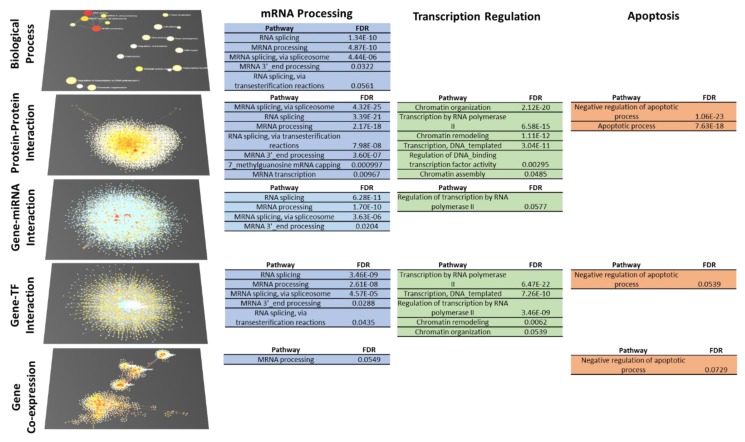
Biological process level systems biology analysis to identify families of processes potentially perturbed by 4SC-202 in tumor cells with low stemness. Networks of genes differentially expressed (DEGs) in healthy, non-stem cell clusters representing drug-treated and control samples (Cluster 2 vs Cluster 3, FDR < 0.05) and interactors were generated at multiple systems biology levels and then the networks were tested for overrepresentation of biological processes. Families of terms were manually curated based on biological knowledge as well as overlap in the gene sets. In the networks, red coloring indicates high significance (biological process) or log fold change values of differentially expressed genes. White coloring indicates low significance (biological process) or gene/protein indicated to interact with DEGs. Blue squares are miRNA or transcription factors (TF) that are indicated to interact with DEGS.

The topologies of the systems biology networks show some consistency: the gene coexpression network has multiple well-defined subnetworks (6 with at least 20 nodes), but the protein, TF, and miRNA networks are all principally composed of a single dense network (Figure 6, left, Figures S8–S11). At the biological process level, the GO term connectivity is generally poor, though some connectivity is shown between the mRNA processing terms (Figure S7). Despite the limitations in this GO network, the consistency across the topologies of the protein, TF, and miRNA networks indicates some complementarity across the different systems biology levels. This complementarity is useful for understanding the mechanism of action of 4SC-202 by identifying trends in the functionalization of these bioelements and their networks.

A functional analysis of our multi-omics network identifies three families of processes that persistently show up across multiple systems biology levels: mRNA processing, transcription/chromatin regulation, and apoptosis-related processes (Figure 6). Terms from each of these families appear in three networks for the transcription/chromatin regulation and apoptosis terms, and in all five networks for mRNA processing terms. In the case of the apoptosis-related terms, three out of four of the terms are negative regulation of apoptotic process. Of the differentially expressed genes mapped to the negative regulation of apoptotic process term, 32 of 39 are upregulated. Consequently, these results do not explain the mechanism by which 4SC-202 decreases the viability of ATRT models.

To confirm this systems-level analysis, a second integrative approach to identify changes to the systems biology landscape was employed. The above-mentioned networks plus the drug-protein networks were subsequently trimmed, integrated, and gene ontology enrichment run on regions that were densely connected. Because processes are connected at different systems biology levels, these densely connected regions may represent areas of one or more related biological processes. Here again, while the gene list in the analysis includes projection and hence may not be entirely accurate, the strength of the analysis is dependent on consistency in the interactions between networks. Gene ontology enrichment results were returned to show the connection between these densely connected regions and hierarchies of biological processes.

**Figure 7:**
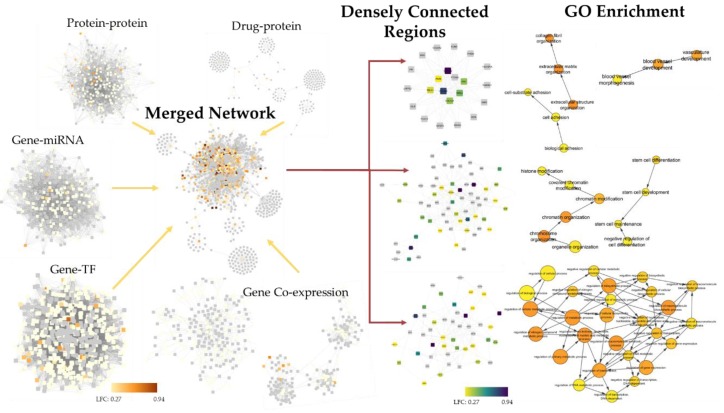
Integrated systems biology analysis to identify biological processes potentially perturbed by 4SC-202 in tumor cells with low stemness. Networks were generated from existing databases at 5 separate systems biology levels from genes differentially expressed (FDR < 0.05) in the “healthy” 4SC-202-treated cluster 2 relative to the ‘healthy’ control cluster 3, trimmed to under 1000 nodes, and then integrated. Densely connection subregions were identified, and GO enrichment was performed to identify “families” of biological processes in the GO hierarchy. Only select GO terms are shown—additional terms are visualized in Figures S12–S16. Grey squares represent genes that are not differentially expressed in the dataset. Colored squares indicate the log fold change of the differentially expressed genes. Coloring of GO circles indicates significance where red is high and yellow is low and the size of the circles indicate how many differentially expressed genes were mapped to each term.

Based on the results from the three most densely connected regions, potentially perturbed biological processes include transcription and histone/chromatin modification (Figure 7). The enrichment of these processes is consistent with the process-level systems biology approach. Additionally, cell-extracellular matrix and extracellular matrix pathways, stem cell-related pathways, blood vessel development pathways, and metabolic pathways appear to be perturbed, which may collectively influence tumor growth, metastasis, and the efficacy of conventional therapies in vivo. Detailed enrichment results from the first five densely connected regions enrichment are available in the Supplemental Information (Figures S12–S16).

A bulk RNA sequencing experiment was also run to validate the single cell results. Because of the high population of unhealthy cells in the single cell dataset, the differential expression of cluster 2 (principally 4SC-202-treated) was taken with respect to clusters 3 and 6 (principally control). Results from this single cell differential expression analysis were compared with results from the bulk RNA-sequencing differential expression analysis between 4SC-202-treated and untreated spheroids. Of the 74 differentially expressed genes from the bulk RNA-sequencing experiment, 25 overlap with the differentially expressed gene from the single-cell RNA-sequencing results (Figure S17a). These overlapping genes are principally structural components of ribosomes and are suitably involved in translation, translocation of proteins, and catabolic mRNA biological processes (Figure S17b,c). These biological processes are also observed in the first functional annotation cluster of terms after separately analyzing each differentially expressed geneset in DAVID (Files S2,S3) [44,45]. These results suggest consistency between the single cell and the bulk sequencing results at the pathway level, though there are only moderate levels of consistency at the gene level.

Collectively, these processes give a starting place for the investigation of a drug mechanism while providing some insight into further areas of potential action, such as metastasis (cell-extracellular matrix interactions), for 4SC-202.

## 3. Discussion

Transcriptomic analysis demonstrates upregulation of epigenetic targets such as class I HDAC’s and LSD1 in 17 age-matched ATRT human tumors as compared to normal brain samples [30]. This upregulation of the Class I HDAC’s and LSD1 in ATRT tumors suggests that 4SC-202 may be an important therapeutic to study in ATRT since 4SC-202 inhibits the Class I HDAC’s as well as LSD1. Indeed, proliferation and viability assays using several different techniques suggests that 4SC-202 is both cytotoxic and cytostatic in two- and three-dimensional cell culture models. In a number of different cancer models studies, 4SC-202 exerts both cytotoxic and cytostatic effects and arrested cell growth in the G2M phase [46]. In this study, 4SC-202 exerted cytostatic properties in the ATRT three-dimensional scaffold model, confirming results obtained in the two-dimensional cell culture.

Standard monoloyer cultures are well established and are easy to use. However, they are characterized by significantly reduced cell-cell interactions, lack of cell-ECM interactions and lack of in vivo-like architecture, diffusion or drug resistance. Spheroids, on the other hand, better recapitulate cell-cell interactions and in vivo-like architecture. However, spheroids showed limited in vivo-like drug resistance, are less amenable for high-content screening, and their size variability affects their reproducibility. In contrast, 3D scaffolds recapitulate key cell-cell and cell-ECM interactions, mimic in vivo-like architecture and complexity, are amenable to high-content screening and recapitulate diffusion gradients of drugs, oxygen, nutrients, and waste, and in vivo-like drug resistance. However, there are still some limitations related to the variability among scaffold matrices (batch-to-batch, animal origin, structure or pore size) [47,48,49].

Higher concentrations of 4SC-202 were needed to exert its cytotoxic and cytostatic effect in the three-dimensional cell culture as compared to in the two-dimensional cell culture. Indeed, a number of studies have shown that 3D scaffolds model recapitulate in vivo-like drug resistance and higher doses might be needed in order to see clinical therapeutic efficacies. The higher drug resistance in 3D scaffolds are likely due in part to the presence of cancer cell-extracellular matrix (ECM) interactions [50,51,52], matrix stiffness [53,54], and concentrations gradients inside 3D scaffolds [55], which collectively affect drug resistance [56]. Furthermore, a spatiotemporal distribution analysis in the 3D scaffolds revealed that 4SC-202 changes the cells’ clustering relative to no treatment by increasing the relative number of non-clustered cells (Videos S1–S4). Previously, treatment with HDAC inhibitors have shown effects on cell-cell, cell-ECM interactions or adhesion/migration mechanisms, which could explain the lack of clustering with a relatively higher number of isolated cells among treated cells [35,36,37].

A number of studies support that 4SC-202 exerts cytotoxic effects by inducing apoptosis in established hepatocarcinoma cell lines and patient-derived cells, colorectal cancer cells, Myelodysplastic Syndrome cells, and human medulloblastoma cells [15,16,17,57], and combined apoptosis and necrosis pathways in urothelial carcinoma cells [13]. The results from the systems biology analysis indicate that genes involved in the negative regulation of apoptosis may be overexpressed. The single-cell RNA-sequencing of the drug-treated cells was performed on the cells that survived 4SC-202 treatment. It is possible that the drug does not cause an enrichment of this pathway but may selectively decrease the viability of cell populations with low expression of genes involved in the negative regulation of apoptosis. Surviving cells may develop cellular mechanisms to be resistant to cell death and apoptosis or were naturally more resistant. Further study is warranted to determine the effect of 4SC-202 on apoptosis pathways and regulation in ATRT.

In addition to the cytotoxic and cytostatic effects of 4SC-202, we found that in a 3D scaffold model of ATRT, 4SC-202 decreases the number of cells expressing CD133, FOXM1, and SOX2, and 4SC-202 decreases the SOX2 population in a spheroid model. While the population diversity of cell lines is limited relative to tumor samples, multiple cancer cell lines have been shown to have a population of cells that mimic cancer stem cell properties including overexpression of cancer stem cell markers, self-renewal, radio-resistance, and metastatic activity [58,59,60,61]. In ATRT, studies have identified a population of cells expressing CD133 with cancer stem cell-like properties that are also resistant to ionizing radiation and have increased radiosensitivity [41,42]. FOXM1 is also closely tied to cancer cell stem cell properties such as cell proliferation, self-renewal, and tumorigenesis [62,63,64,65,66]. In glioblastoma, inhibition of FOXM1 sensitized tumors to irradiation in vitro and in vivo [67]. SOX2 is another marker of cancer cell stemness in multiple cancer types [68,69,70]. Because 4SC-202 affects the cell population(s) that express CD133, FOXM1, and SOX2 and there is strong evidence that these proteins are cancer stem cell markers, our data suggest that 4SC-202 may decrease the cancer stem-like cell population. Our studies do not show whether this decrease is due to the death or transformation of the stem-like cell population, however, having a therapeutic such as 4SC-202 that affects a cell population with high expression of stem cell markers may be highly beneficial as an adjuvant since it could reduce metastasis or resistance to standard of care treatments, such as radiation.

The exact mechanism by which 4SC-202 affects the stem-like cell population is not fully investigated by this study, however, the overexpression of both FOXM1 and SOX2 in the control single cell population with stem-related features is suggestive that changes to the stem-like cell population may be affected by influencing the expression of these proteins. In glioblastoma, FOXM1 has been shown to promote stemness by modulating SOX2 expression in vitro and in vivo [67], and in neuroblastoma cells, FOXM1 was shown to directly activate SOX2 expression [71]. After treatment with a dual HDAC and PI3K inhibitor in high grade pediatric glioma, the expression and transcriptional activity of FOXM1 decreased [72], which is consistent with our finding that FOXM1 expression was altered.

FOXM1 has also been used as an indicator of MYC transcriptional regulation in ATRT [73] suggesting that changes in the MYC pathway may influence FOXM1 expression. FOXM1 was also identified as the downstream target of MYC in prostate cancer [74]. This relationship between MYC and FOXM1 is of particular interest since MYC is one of the key distinguishing features of the ATRT-MYC type, and the cell line used for the single-cell RNA-sequencing experiment has recently been classified as ATRT-MYC type [31,32]. Inhibition of MYC has been demonstrated to decrease pluripotency-related pathways and tumor self-renewal [73,75], and is altered by HDACi’s. In a model of acute myeloid lymphoma, HDACi’s SAHA and MS27-275 acetylate MYC, decreasing its expression [76]. MYC-induced transcriptional pathways were also found to be reactivated after treatment with HDACi’s in hematologic malignancies [77]. Collectively these results suggest that further investigation into the effect of 4SC-202 on MYC, FOXM1, and SOX2 may clarify the mechanism by which 4SC-202 is decreasing the stem-like population in these 3D models of ATRT.

Since the extracellular matrix plays an important role in activating endothelial cells and inducing proliferation, migration, and angiogenesis [78], the extracellular-matrix- and angiogenesis-related pathways identified by the integrated systems biology may have been perturbed by 4SC-202-treatment. Several members of the HDAC family have been shown to be involved in the regulation of genes in the extracellular matrix and tumor cells that influence angiogenesis [79]. Additionally, there are results suggesting that other HDACi’s influence metastasis, but whether as a promotion or inhibition is dependent on the study [80,81,82,83]. 4SC-202–induced inhibition of HDAC’s may therefore play an important role in metastasis and/or angiogenesis.

Other pathways potentially perturbed by 4SC-202 include mRNA regulation, transcription, and histone/chromatin organization, which are closely related to the histone-level mechanism of action of 4SC-202. Changes to these pathways are consistent with expected results and support the validity of the systems biology analyses. The systems biology analyses also suggested that biosynthetic and metabolic pathways may have been perturbed. These biological processes may warrant further investigation to fully understand the mechanism of action of 4SC-202 in ATRT.

This is the first study to examine the single-cell heterogeneity in ATRT following treatment with a newly developed dual HDAC LSD1 inhibitor and the effect of the drug on the genomic landscape. The findings that 4SC-202 reduces the stem cell population as assessed by single cell RNA sequencing and the potential use of a targeted therapy in ATRT are unique and will have important implications for the treatment of ATRT.

## 4. Materials and Methods

### 4.1. Cell Culture

The human cell line CHLA-06-ATRT denoted in this manuscript as ATRT-06 and CHLA-05-ATRT (ATRT-05) were obtained from the posterior fossa of a 4-month-old female patient and the frontal lobe of a 2-year-old male patient, respectively. ATRT-06 was purchased from ATCC (Manassas, VA, USA, CRL-3038). Control cells include neural stem cells (Lifelinebiotech, Reno, NV, USA) and astrocytes and were cultured according to manufacturer’s protocols. Cancer cells and HEK-293 were cultured in DMEM with 10% FBS, penicillin/streptomycin, and amphotericin, and incubated at 37 °C and 5% CO_2_. Spheroids were formed by plating 1000 cells in culture media per well in ultra-low attachment Corning spheroid microplates.

### 4.2. Spheroid Model

Cells were plated in triplicate in 96-well ultra-low attachment plates (Corning, Corning, NY, USA) at 1000 cells-well at the indicated concentrations of 4SC-202, media alone, or DMSO control (0.02%). Seven days following plating of cells, spheroids were treated with 4SC-202 (Selleckchem, Pittsburgh, PA, USA) at concentrations ranging from 1 nM to 50 µM and corresponding DMSO control (0.02%) for 72 h prior to be analyzed for cytotoxicity with the Sytox Green (Invitrogen, Carlsbad, CA, USA) or dissociated and frozen for RNA sequence analysis.

### 4.3. Development of 3D Scaffolds to Study the Effect of Domatinostat (4SC202) on ATRT-06 Cell Survival

ATRT-06 cells (1 × 10^6^ cells/mL) were pre-labeled with DiO (10 μg/mL) for 1 h. Then, DiO-labeled ATRT-06 cells were embedded in a 3D matrix scaffold that was engineered through the cross-linking of fibrinogen into fibrin as previously described [55,84,85]. Briefly, a mixture of plasma, ATRT-06 cell suspension (1.5 × 10^5^ cells/per scaffold) in DMEM complete media was embedded into scaffolds that were then crosslinked using calcium chloride (CaCl_2_) and stabilized with trans-4-(Aminomethyl) cyclohexanecarboxylicacid (AMCHA). ATRT-06 cells grown within the scaffold were treated with Domatinostat (4SC-202) at a range of concentrations (0–50 μM). The treatment was carried out for 3 days during which the 3D ATRT-06 scaffolds were incubated either in 96 well plates or in 8-well chamber slides for confocal imaging. 3D scaffolds containing ATRT-06 cells incubated with DMEM complete media or DMSO were used as controls.

### 4.4. Sytox Green Cell Proliferation Assay and Viability Assays in 2D

1000 cells were plated per well of a 96-well white plate (Corning) and allowed to attach overnight before being treated with 4SC-202 or vehicle DMSO at concentrations ranging from 0–20µM. After 72 h of treatment, cells were stained with Sytox Green (Thermo Scientific, Carlsbad, CA, USA) to measure dead cell fluorescence, then permeabilized with 0.6% Triton X to determine live and total cell fluorescence. Cell titer Glo Luminescence viability assay (Promega, Madison, WI, USA) was also performed as an alternate method to verify viability.

### 4.5. Flow Cytometry for Spheroid Analysis

Cells were washed with 1X PBS and blocked with 2% FCS. For staining with CD133 (Miltenyi Biosciences, Bergisch Gladbach, Germany, cat# 130-080-801), the antibody and the isotype control were added separately to live cells and stained at 40 °C. For staining other antibodies, cells were fixed using the 1:3 diluted Fix: Perm solution (Ebioscience, Carlsbad, CA, USA, cat# 00-5523-00) for 30 min at room temperature. These cells were then washed with the Perm buffer (Ebioscience, cat# 00-5523-00). Appropriate amounts of SOX2 antibody (Ebiosciences, cat# 50-9811-82) and isotype control were added to separate tubes with fixed cells for 30 min at room temperature. The cells were washed with PBS and analyzed using the BD ARIA (BD Bioscience, San Jose, CA, USA) cytometer. Flow cytometric plots were obtained with Flow Jo (version v9/10). All the analyses were made with a starting count of 1 million total viable cells.

### 4.6. Flow Cytometry for 3D Scaffold Analysis

At day 3, 3D scaffolds were enzymatically digested with collagenase (20 mg/mL for 2–3 h at 37 °C), ATRT-06 cells were retrieved and analyzed. ATRT-06 cells were isolated and identified by gating cells with a high DiO signal (excitation, 488 nm; emission, 530/30 nm). Cell viability was evaluated by using a Sytox Blue live-dead fluorescent dye (S34857, Invitrogen) possessing excitation, 358 nm, emission, 461 nm. For all analyses, a minimum of 10,000 events was acquired using BD FACS Fortessa and FACSDiva v6.1.2 software. The ATRT-06 cell counts were always normalized to a predetermined number of counting beads (424902, Biolegend, San Diego, CA, USA), and the data was analyzed using FlowJo v10 (FlowJo, Ashland OR, USA).

### 4.7. Immunohistochemistry (IHC) and Immunofluorescence (IF) Staining of 3D Scaffolds

3D scaffolds containing ATRT-06 cells were fixed in 10% neutral buffered formalin and processed on a Leica 300 ASP tissue processor. Paraffin-embedded matrix sections were longitudinally sliced at 10 μm. The BenchMark^®^ XT automated slide staining system (Ventana Medical Systems, Inc., Oro Valley, AZ, USA) and the Ventana iView DAB detection kit was used as the chromogen, and the slides were counterstained with hematoxylin and eosin (H&E). H&E images were imaged using an Aperio VERSA Bright field Fluorescence & FISH Digital Pathology Scanner (Leica, Westwood, NJ, USA). For IF studies, paraffin sections were dewaxed in the following order: 10 min in xylene, 10 min in 100% ethanol, 10 min in 95% ethanol, 10 min in 70% ethanol and 10 min in distilled water, followed by rehydration in wash buffer (0.02% BSA in PBS) for 10 min. After this, sections were subjected to incubation in blocking buffer (5% BSA in PBS) for 60 min at room temperature to block non-specific staining between the primary antibodies and the sample. Sections were rinsed with washing buffer and incubated in incubation buffer (1% BSA in PBS) with different primary antibodies. Primary antibody incubation was carried out overnight at 4 °C to allow for the optimal binding of antibodies to sample targets and reduce non-specific background staining. The following primary antibodies: anti-CD133 (11-1339-42, 1:100, eBioScience, San Diego, CA, USA), anti-FOXM1 (sc-376471, 1:100, Santa Cruz Biotechnology, Santa Cruz, CA, USA), and anti-SOX2 (ab97959, 1:100, Abcam, Burlingame, CA, USA) were used. Wherever applicable, FITC conjugated (SAB4600042, 1:1000, Sigma Aldrich, Saint Louis, MO, USA) and Alexa Fluor 594 conjugated (A11037, 1:1000, Thermo Fischer Scientific, Waltham, MA, USA) secondary antibodies were used. For all the samples, blocking and incubation buffers were prepared in 1X permeabilization buffer (Biolegend). The dilution of antibodies was carried out accordingly to the manufacturer’s instructions. Lastly, a drop of anti-fade mounting media containing DAPI was added to the samples and sections were imaged.

### 4.8. Confocal Imaging of 3D Scaffolds

3D scaffolds containing DiO-labeled ATRT-06 cells growing in 8 well chambers and paraffin section matrix slides after IF staining were imaged using a Nikon Ti2-A1TR confocal microscope (×20 dry, ×40 oil and ×60 oil objectives, 2.5 magnified) and analyzed using NIS elements software (Nikon, Melville, NY, USA). Samples were excited at 488 nm (FITC/DiO), 358 nm (DAPI), 540 nm (Alexa Fluor 594), and the emission light was collected at 500–530 nm, 461 nm, and 620 nm long pass, for each channel, respectively. Z-stack images of approximately 0.5 mm thickness were taken for each sample at 2 μm step sizes. Each frame consisted of a 520 × 520-pixel image, taken at a rate of 1 μs/pixel. Captured sample images were manually analyzed using the Nikon NIS-Elements Advanced Research version 3.2 software (Nikon Instruments, Tokyo, Japan) according to the manufacturer’s guidelines. Briefly, from the total scan of each captured image, a region of interest (ROI) was selected using a 10× magnification at a resolution of 1280 × 1024 (RGB 8 bit). FOXM1, SOX2, CD133 and DAPI were visible in red, red, green and blue channels respectively, and each corresponding channel was selected to perform a total cell number count for each marker. An additional deconvolution step was performed to remove maximum unwanted noise. The object count option on the software was utilized to count the cell numbers in the ROI by manually tagging each cell in each channel. These steps enhanced the discrimination ability of the software by using uniform parametric thresholds and by also improving the signal/noise ratio. The object thresholds were manually adjusted to remove artifacts and to gather or entirely distinct closed detected objects.

### 4.9. Cell Clustering Analysis of Confocal Images From 3D Scaffolds

DiO- and DAPI-labeled confocal micrographs were batch converted to TIFF stacks using Fiji’s (version 2.0.0-rc-69/1.52p) batch converter [86,87]: no interpolation, scale factor 1.00, and read using Bio-Formats version 6.3.1 [88]. Using a custom R (version 3.6.0) script [89,90,91,92], each TIFF stack had its slice intensities summed into two values per slice: sum DAPI intensity and sum DiO intensity. As part of image pre-processing, the sum intensity outliers were determined using Tukey’s “inner fences” method [93]. The highest slice with either DAPI or DiO absolute maximum intensity was also determined. Any slice that was both higher than the slice of absolute maximum intensity and had an outlying sum intensity was removed from the rest of the stack (Figure S18). The final minimum slice value acts as a dividing line between where the fluorescence signal represented cells on top of the glass (2D cell culture) and the cells growing free in the scaffold (3D cell culture). The cropped TIFF stacks were saved as separate channels for subsequent pre-processing.

The following operations were performed using a custom macro in Fiji [86,87]. The cropped channel images were duplicated, and threshold values were retrieved via the auto-threshold function using Otsu’s method [94,95]. Object data was extracted using 3D Objects Counter [96]: show masked images with redirection to other duplicate images, threshold value retrieved from Otsu’s auto-threshold, including object sizes from ten pixels to maximum volume, exclude objects on edges, show statistics and summary. The masked images were saved as TIFF stacks, and object data statistics tables were saved as CSVs for use in R.

Using a custom R script (R Core Team, 2016), DiO object volume data (in µm^3^) was compiled according to condition and day. Density histograms (Figure S3) were generated with the “ggpubr” and “scales” R packages. The “peak region” histograms have domains [0, $\bar{x}$ + 2*σ*], where the sample mean and standard deviation are derived from the respective histogram’s data.

Using a custom macro in Fiji [86,87], each condition and day’s channels were merged accordingly, made into composites, and had their objects smoothed. Resultant image stacks were saved as TIFFs in preparation for 3D videos. The composite images were manually viewed using Fiji’s 3D Viewer version 2.0 [97]: threshold 0, resampling factor 1, all channels displayed. Bounding boxes and coordinate systems were shown. An appropriate view angle was manually chosen, and the animation options were changed such that each image was rotated around the y-axis, and the rotation interval was 1.00 degree. Videos were recorded of a 360-degree rotation, and each video was saved as an AVI with JPEG compression and 24.0 fps framerate. Day three videos are available as Videos S1–S4.

### 4.10. Single-Cell RNA-Sequencing Experimental Protocol

The ATRT cell line ATRT-06 spheroids were grown in cell culture (DMEM, 10% FBS, Penicillin/Strep) at 37 °C. The cells were initially harvested using 0.25% Trypsin-EDTA, and a cell count performed using Trypan blue exclusion assay. 1000 cells were plated per 100 microliters of medium in a 96 well of an ultra-low attachment Corning spheroid microplate.

On day 7 after plating, spheroids were assigned to one of three experimental groups: early untreated (small spheroid group), vehicle-treated control (large spheroid), and 4SC-202-treated (large spheroid) groups. Early smaller untreated spheroids were dissociated on day 7 with Accumax (Millipore Sigma, St. Louis, MO, USA). Approximately 10,000 dissociated ATRT-06 cells were resuspended in a resuspension medium containing DMEM with 40% FBS and then 5,000 live cells were frozen into cryovials containing DMEM with 30% DMSO and 40% FBS as described in the Cryopreservation Protocol for Cryopreservation of Human PBMC’s in the 10× Genomics protocol.

Remaining spheroids were treated either with 56 nM 4SC-202 (4SC-202-treated group) or 0.02% DMSO (vehicle-treated control group) for 72 h. On day 9, 4SC-202-treated and vehicle-treated spheroids were dissociated and frozen down as described above. Approximately 20,000 cells were dissociated from ATRT-06 spheroids for each control and experimental group. Following dissociation of the spheroids with Accutase, cells were stained with Trypan Blue and counted using the Countess (Invitrogen). 10,000 live cells from 4SC-202-treated or vehicle-treated control spheroids and were then frozen using DMEM with 30% DMSO and 40% FBS (10× Genomics).

Library preparation, sequencing, and the initial run of Cell Ranger pipeline were performed at the University of Minnesota Genomics Center. After thawing, the cells were counted, and the percent viability calculated. The early untreated experimental group had 4,500 cells with 60% viability, the vehicle-treated group had 9,500 cells with 63% viability, and the 4SC-202-treated experimental group had 8,000 cells with 72% viability. The 10× Genomics libraries were prepared using the Single Cell 3’ v2 chemistry. Libraries were sequenced on an Illumina HiSeq 2500 in High-output mode (paired-end, index: 8 bp, read 1: 26 bp, read 2: 98 bp). Minimum intended coverage was 25,000 reads per cell. Actual coverage was 244,859 reads/cell for early untreated control spheroids (harvested day 7), 545,618 reads/cell for vehicle-treated spheroids (harvested day 9), and 19,353 reads/cell for 4SC-202-treated spheroids (harvested day 9).

### 4.11. Single-Cell RNA-Sequencing Data Analysis

10× Genomics single cell data were demultiplexed, aligned, and quantified using the Cell Ranger pipeline (version 1.3.1, aligned to GRCh38). 10× data were read into the Seurat workflow for quality control, integration, clustering, and differential expression analysis (version 3.0.1) [36,37]. Cells with more than 40,000 RNA molecules were trimmed before further analysis (Figure S19A). Data were normalized and variable features identified for individual samples. 4000 rather than the default 2000 variable features were identified in order to increase the number of features included with low but significant expression changes (Figure S19B–D). Integration steps were performed with 50 dimensions. The first 50 principle components (PCs) are used for inputs into the Shared Nearest Neighbor Graph for clustering and the Uniform Manifold Approximation and Projection (UMAP) algorithm based on the variability of the datasets. All of the first 50 PCs are considered significant relative to a uniform distribution of p values, though the variance explained by each additional PC appears to be small by 50 (Figure S19E,F). Graph-based clustering was performed with Seurat workflow without user explicitly choosing number of clusters. Seurat clustering was validated by comparing with default clustering in the Cell Ranger pipeline (Figure S20).

Differential expression was run based on a Wilcoxon Rank Sum test (|Log Fold Change| > 0.25, *p* < 0.01) or by using a Poisson generalized linear model. Results from the Poisson model were used exclusively for comparison with the bulk sequencing differential expression. Differentially expressed genes from the Poisson differential expression were also input into DAVID [44,45] for functional annotation analysis and clustering with a Homo sapiens background. *p* values were adjusted using the Bonferroni correction for multiple testing. To confirm results from the Seurat workflow, or in cases where interesting features are excluded due to the gene selection step in the Seurat workflow, locally distinguishing significant feature comparisons were run on Loupe Cell Browser and/or an ANOVA with a post-hoc Tukey’s Honestly Significant Difference test were run in R using base packages and dplyr. Features were normalized according to the CellRanger protocol prior to testing in Loupe Cell browser and were scaled by a size factor that was calculated as the total UMI count per cell divided by the median UMI sum across all cells prior to ANOVA testing. Raw single-cell RNA-sequencing data were submitted to the Sequence Read Archive under the BioProject PRJNA588272.

### 4.12. Bulk RNA-Sequencing

Approximately 10 days after plating, spheroids were treated with 56 nM 4SC-202, vehicle (DMSO 0.02%), or media only for 72 h. RNA extraction (RNeasy, Qiagen, Germantown, MD, USA) was performed on the spheroids and RNA quality was assessed using BioAnalyzer. The samples were sent to the Genomics Sequencing Facility at South Dakota State University for library preparation and sequencing. The samples were sequenced using Illumina NextSeq 500 (paired-end, 76 bp, 4 lanes). FASTQ files were checked for quality control via BaseSpace. Files were uploaded as pair-end reads to CLC-Bio and reads were aligned to GRCh38.p13. Gene quantification was performed with default options. Differential expression analysis was performed using TPM values in OmicsBox using edgeR with default parameters [98]. Differentially expressed genes (FDR < 0.05) were input into DAVID [44,45] for functional annotation analysis and clustering with a Homo sapiens background. Raw sequencing reads are available on the Sequence Read Archive under the BioProject PRJNA600953.

### 4.13. Microarray Analysis

Normalized gene expression data from Affymetrix HG-U133 Plus 2.0 arrays were downloaded from Gene Expression Omnibus (GEO) repository number GSE35493 [29]. Of the samples, *n* = 2 were normal pediatric cerebellum tissue, *n* = 9 were normal pediatric brain tissue, and *n* = 20 were ATRT tissue. The normal brain tissue included *n* = 2 pediatric cerebellum, *n* = 2 pediatric occipital lobe, *n* = 2 pediatric parietal lobe, *n* = 2 temporal lobe, and *n* = 1 pediatric frontal lobe tissue samples. All Affymetrix probesets mapped to the genes of interest were considered. Error bars represent the standard error of the mean. Significance was determined using the Linear Models for Microarray Data (LIMMA) package implemented in GEO2R [99]. P values were adjusted using the Benjamini and Hochberg procedure.

### 4.14. NanoString Analysis

Raw gene expression data from the NanoString nCounter technology that were previously published in Chakravdhanula et al. [30] were reanalyzed using the NanoStringBioNet workflow [100]. Of the samples, *n* = 7 were age-matched patient-derived neuronal samples and *n* = 17 were ATRT tumor tissue. All samples were frozen tissue samples. Normalization of the expression data was performed using the NanoStringQCPro package version 1.16.0 [101]. Differential expression analysis was performed using NanoStringDiff version 1.14.0 [102]. Normalized data are available upon reasonable request.

### 4.15. Biological Process-Level Systems Biology Analysis

Genes with an adjusted p value of less than 0.05 were considered differentially expressed. Log fold changes for differentially expressed genes were input into NetworkAnalyst 3.0 [43,103] for network generation. Cerebellum-specific protein–protein interactions (ppi) come from the DifferentialNet database (filter 15.0^th^ percentile) [104], gene-transcription factor interactions are based on the ENCODE ChIP-seq data [105], gene-miRNA interactions are from TarBase and miRTarBase [106]. The cerebellum gene co-expression network is based on data from the TCSBN database [107]. For each network, overrepresentation analysis (ORA) was performed on each network compared to the gene sets in the PANTHER: BP database and processes with an FDR < 0.1 were considered significant. ORA results from the gene set, cerebellum ppi network, gene-TF network, gene-miRNA network, and cerebellum co-expression networks were pooled and tabulated, and terms that appeared in more than three networks were further assessed to see if related terms also appeared. The terms “Skeletal system development”, “Protein localization”, and “Viral process” appeared in at least three networks but were dismissed because they were isolated terms and were of less biological interest.

### 4.16. Integrated Systems Biology Analysis

The ppi, gene-TF, gene-miRNA, and co-expression networks that were created as described above were trimmed in NetworkAnalyst to between 200 and 600 nodes (200 to 17,000 edges) by increasing the degree or betweenness requirements (ppi: 10 degree, gene-TF: 25 degree, gene-miRNA: 25 degree, gene co-expression: 2 degree, 4 betweenness). A protein-drug interaction network was also created based on data collected from DrugBank 5.0 [108]. All of the trimmed networks were merged in Cytoscape [109]. In the merge, 283 nodes were replicates, but only 20 edges suggesting that the networks were largely non-redundant. Clusters of terms were identified from the merged network using the MCODE application with default settings [110]. Clusters were then tested for GO biological processes overrepresentation using the BiNGO application [111].

## 5. Conclusions

ATRT is an aggressive pediatric cancer that relies upon a standard of care that has lasting and severe side effects. In this study, 4SC-202 was examined as a potential small molecule therapeutic to ATRT in 2D and 3D cell culture models. 3D cell culture models included spheroids and a plasma-based scaffold that mimics in vivo extracellular matrix conditions. We found that targets of 4SC-202, *HDAC1*, *HDAC2*, *HDAC3*, and *LSD1* are significantly overexpressed in ATRT relative to normal brain. In both 2D and 3D cell culture models, 4SC-202 had cytostatic and cytotoxic effects. Single-cell RNA-sequencing of the spheroid model of ATRT indicated that a population of cells that overexpressed stem-cell-related genes was significantly reduced after treatment with 4SC-202. This population of cells overexpressed both *SOX2* and *FOXM1*, which interact in the protein–protein network. In both the spheroid model and the scaffold model, the SOX2 high population was shown to decrease with 4SC-202-treatment, and in the scaffold model, FOXM1, and CD133 populations decreased with treatment. These results indicate that 4SC-202 affects a population of cells with stem-like expression profiles. Multiple systems biology analyses were also conducted to identify potentially perturbed pathways in the systems biology landscape of the tumor cell populations from single and bulk RNA-sequencing datasets. Resulting processes included apoptosis regulation-related, extracellular matrix-related, angiogenesis-related, and metabolism-related biological processes. Further studies are warranted to assess the effect 4SC-202 may have on the radio-sensitivity and metastasis of ATRT.

## Supplementary Materials

The following are available online at https://www.mdpi.com/2072-6694/12/3/756/s1, Figure S1: Epigenetic regulators are overexpressed in ATRT relative to normal brain, Figure S2: 4SC-202 reduces growth of 3-dimensional ATRT-06 spheroids, Figure S3: Density plots of cell size from segmented confocal images of ATRT scaffold model, Figure S4: 4SC-202 significantly reduces *HDAC1*, *HDAC2*, and *SMARCB1* gene expression, Figure S5: Stem cell markers are underexpressed in 4SC-202-treated spheroids and overexpressed in cluster 6, Figure S6: Highly differentially expressed genes are stable across comparisons, Figure S7: Enriched biological process network, Figure S8: Cerebellum protein-protein interaction (ppi) network, Figure S9: Gene-miRNA interaction network, Figure S10: Gene-transcription factor interaction network, Figure S11: Cerebellum gene co-expression network, Figure S12: Densely connected subregion (cluster) 1 and biological process enrichment from integrated systems biology analysis, Figure S13: Densely connected subregion (cluster) 2 and biological process enrichment from integrated systems biology analysis, Figure S14: Densely connected subregion (cluster) 3 and biological process enrichment from integrated systems biology analysis, Figure S15: Densely connected subregion (cluster) 4 and biological process enrichment from integrated systems biology analysis, Figure S16: Densely connected subregion (cluster) 5 and biological process enrichment from integrated systems biology analysis, Figure S17: Genes differentially expressed between 4SC-202-treated and control spheroids in both single-cell and bulk RNA-sequencing datasets are enriched for ribosome-related GO terms, Figure S18: Cropping location relative to the total intensity along the z-axis, Figure S19: Parameter selection plots for single cell analysis in Seurat, Figure S20: Seurat graph-based clusters correlate with clusters identified using the CellRanger pipeline, File S2: DAVID functional annotation clustering results for bulk sequencing, File S3: DAVID functional annotation clustering for single cell sequencing, Video S1: Cropped and thresholded confocal image stack from scaffold with no treatment 3 days after seeding, Video S2: Cropped and thresholded confocal image stack from scaffold treated with DMSO 3 days after seeding, Video S3: Cropped and thresholded confocal image stack from scaffold treated with 0.02 µM 4SC-202 3 days after seeding, Video S4: Cropped and thresholded confocal image stack from scaffold treated with 50 µM 4SC-202 3 days after seeding.
