# Supplementary material for: Analysis of Dual Class I Histone Deacetylase and Lysine Demethylase Inhibitor Domatinostat (4SC-202) on Growth and Cellular and Genomic Landscape of Atypical Teratoid/Rhabdoid
Source: Cancers (Basel). 2020 Mar 23;12(3):756. doi: 10.3390/cancers12030756 (PMC7140080; doi:10.3390/cancers12030756)
Supplement: Supplementary file 1 [file cancers-12-00756-s001.zip › cancers-719592 supplementary final/cancers-719592-Supplemental figures final.pdf]

Supplemental Materials:

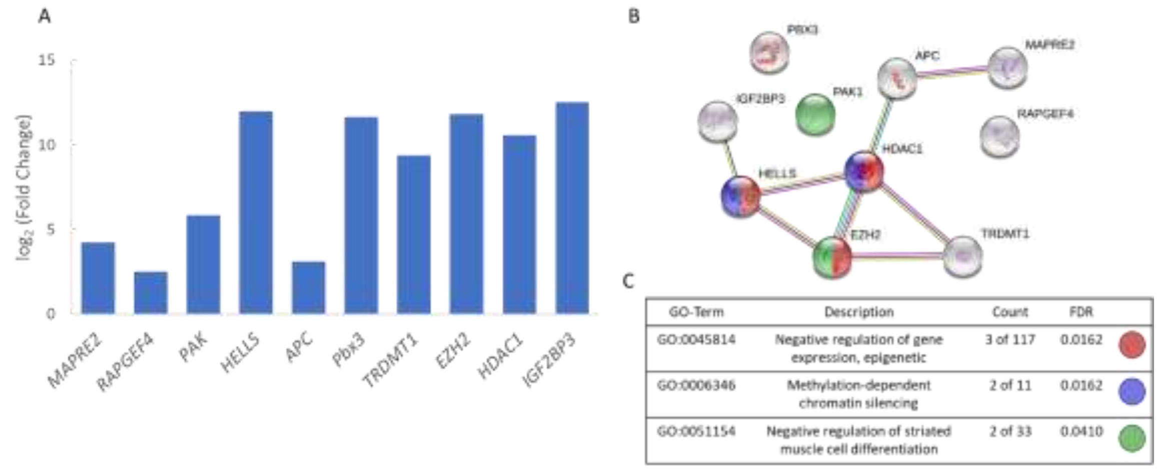

Figure S1. Epigenetic regulators are overexpressed in ATRT relative to normal brain. (A) Log_2 fold changes of the ten most differentially expressed genes (lowest FDR) in frozen ATRT tissue (n = 17) relative to normal brain tissue (n = 7). Gene expression is measured using NanoString technology with a previously published custom gene panel [1]. (**B**) STRING 11.0 protein-protein interaction network of ten genes shows high connectivity relative to a random protein set (p = 0.000175) (**C**) All significant results from gene-set enrichment analysis in STRING (FDR < 0.05). HDAC1, EZH2, and HELLS are implicated in GO terms associated with epigenetic regulation [2].

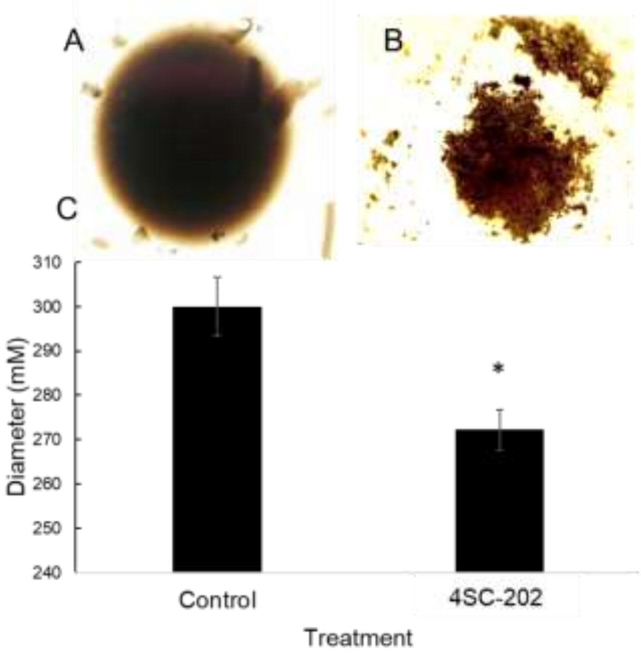

Figure S2. 4SC-202 reduces growth of 3-dimensional ATRT-06 spheroids. (**A**) Spheroids treated with DMSO control (0.02%) are more defined and have significantly increased diameter compared to (**B**) 4SC-202-treated (56 nM) spheroids. (**C**) Diameters of control and treated spheroids. Spheroids were imaged using the Olympus (BX51) and diameters quantified using cellSens (Standard).

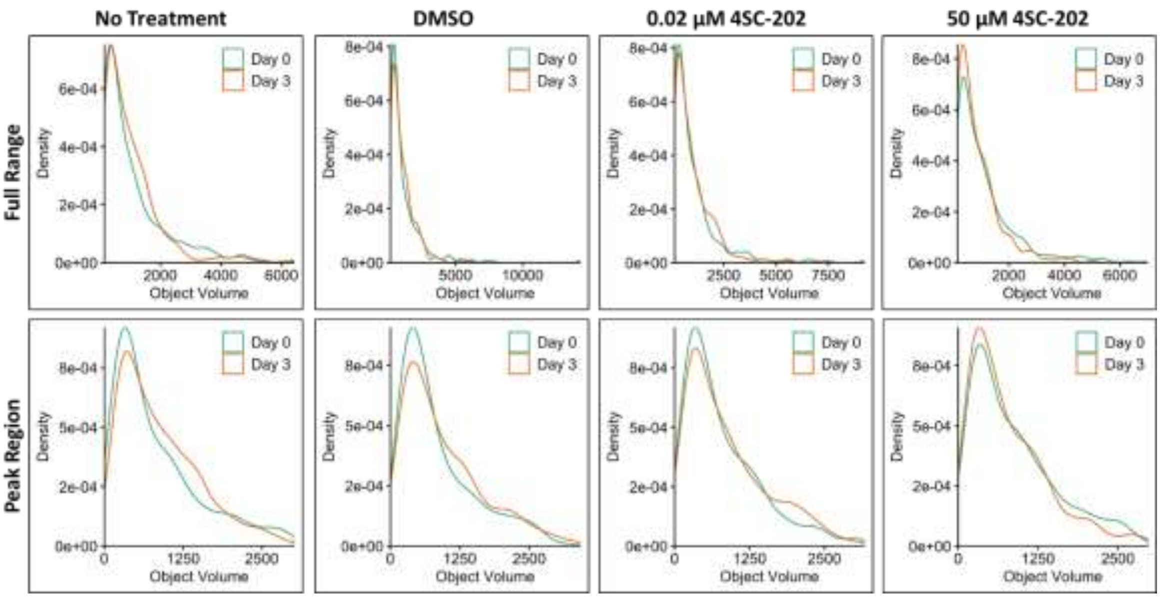

Figure S3. Density plots of cell size from segmented confocal images of ATRT scaffold model. In each of the experimental conditions, the most common segmented volume is between 400–500 µm^3. Based on the approximate cell volume, it is likely that the peak for the absolute maxima represents the population of non-clustered cells. Experimental conditions with high populations of non-clustered cells would also be more likely to have fewer, well defined clusters, which may be represented by the clear substructures in the density plot. In contrast, experimental conditions with a lower population of non-clustered cells and broader peaks are likely to represent models that have higher levels of clustering. The control experimental conditions show a shortening of the central peak from day 0 (blue green) to day 3 (orange) and an increase in the size of the peaks at larger volumes, suggesting that there is an increase in the number and size of cell clusters. The density plot for the 0.02 µM 4SC-202-treated scaffold likewise has a shorter peak on day 3 relative to day 0, but the substructures are less pronounced excepting the peak at around 2000 µm^3. In contrast, for the 50 µM treated scaffold, the central peak is higher in day 3 relative to day 0, suggesting that there is a higher percentage of cells that are isolated or in very small clusters on day 3 relative to the control groups.

A B

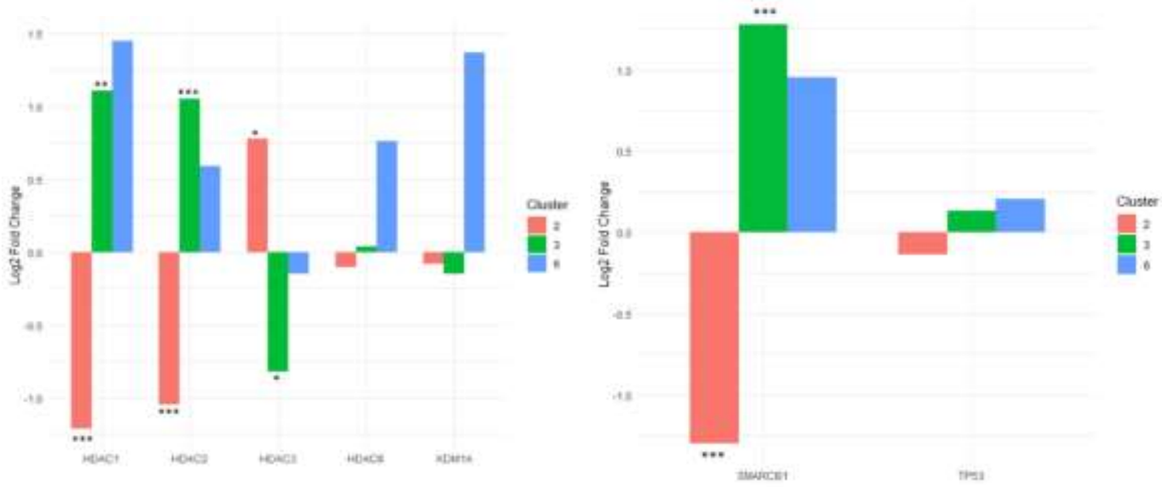

Figure S4. 4SC-202 significantly reduces *HDAC1*, *HDAC2*, and *SMARCB1* gene expression. (**A**) Log2 fold change of the average expression and significance of Class I HDACs and *KDM1A* (LSD1). (**B**) Log2 fold change of the average expression and significance of *SMARCB1* (SNF5/INI1/BAF47) and *TP53* (P53). Log fold changes and significance were exported from Loupe Cell Browser and are based off of a locally distinguishing significant feature comparison of clusters 2, 3, and 6. p value < 0.001 (***), p value < 0.01 (**), p value < 0.1 (*).

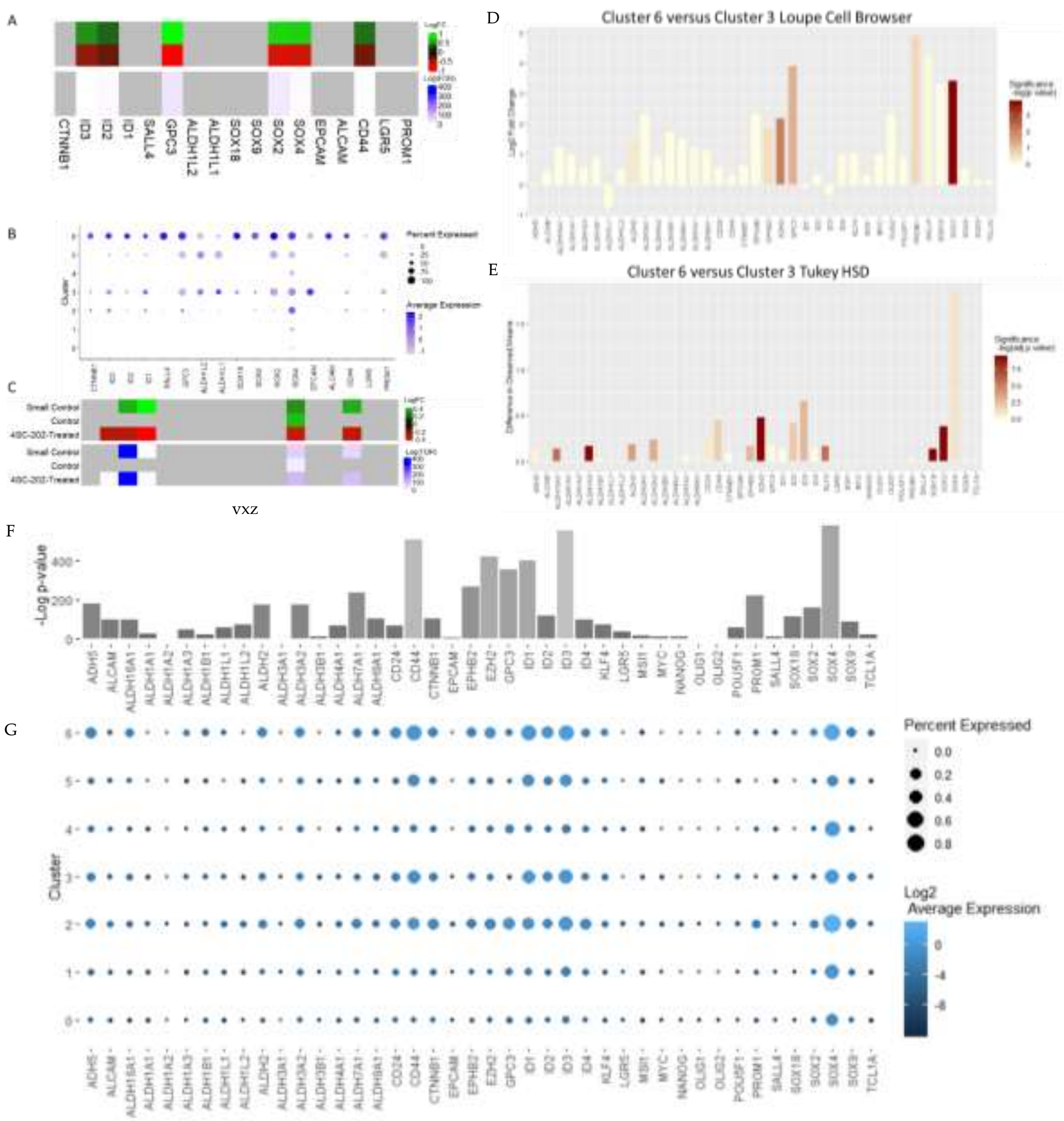

Figure S5. Stem cell markers are underexpressed in 4SC-202-treated spheroids and overexpressed in cluster 6. (**A**) Log_2 fold change (LFC, top heatmap) and significance of stem cell marker expression (bottom heatmap) between cluster 6 and cluster 3. Both clusters are principally composed of control cells that have a low percentage of mtRNA. Significance is calculated using the Wilcox Rank Sum test in Seurat ($p < 0.01$, $|LFC| > 0.25$) [3,4]. Green indicates positive log fold expression changes and red indicates negative log fold expression changes. Non-significant changes are grey. Significance is shown as the negative log of the FDR. No significance is grey, low significance is white, and high significance is blue. Heatmaps are generated with the ComplexHeatmap package in R [5]. (**B**) Dot plot visualization of the average expression and percentage of cells expression stem cell markers or stem cell-related genes across samples generated in Seurat. Color of the dot indicates the average expression of the feature and the size indicates what percentage of the cells express the feature. (**C**) Log_2 fold change and significance of stem cell marker expression between experimental conditions. Heatmaps were generated as described above. (**D**) Log_2 fold change and significance of stem cell-related genes in cluster 6 relative to cluster 3 as calculated in Loupe Cell Browser using locally distinguishing significant feature comparison tool. Color indicates the significance where high significance is red and low significance is tan. (**E**) Difference in observed means and significance of stem cell-related genes based on Tukey's Honest Significant Difference Test between clusters 6 and 3 following an ANOVA of all clusters (see **F**–**G**). Color indicates the significance where high

significance is red and low significance is tan. (**F**) Barplot of the negative log of the significance of an ANOVA test for each stem cell-related gene. Data were scaled by a size factor that was calculated as the total UMI count per cell divided by the median UMI sum across all cells prior to testing. (**G**) Dotplot of average scaled expression and percentage of cells expressing a gene across all clusters. Cells with a gene expression above 0 were considered to express a gene. Color indicates the log2 transformed average expression, where light blue indicates a higher average expression.

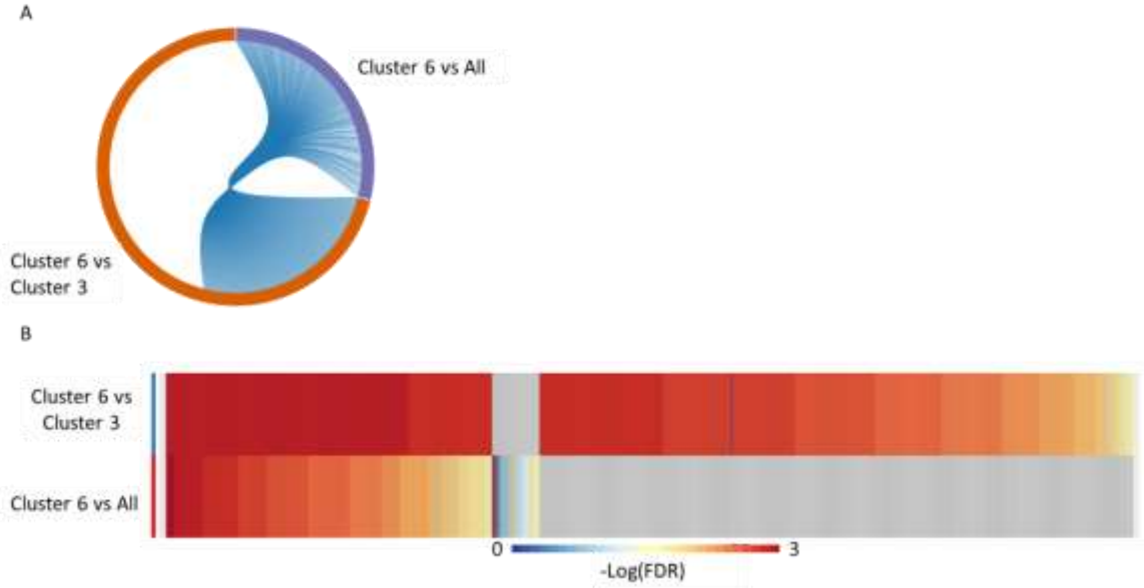

Figure S6. Highly differentially expressed genes are stable across comparisons. (**A**) Circos diagram of the overlap in the differentially expressed genes when comparing Cluster 6 to all cells (control and treated) or to just Cluster 3 (principally control cells). Differentially expressed genes are considered genes that have an FDR < 0.05. (**B**) Heatmap of the –Log(FDR). Heatmap and Circos diagram were generated from differentially expressed genes using NetworkAnalyst [6]. Because Cluster 3 and Cluster 6 are both principally composed of control spheroids, the comparison of these two clusters is close to a comparison of the two populations within the healthy control. Cluster 6 versus all takes into consideration the effect of the drug and includes a comparison with the drug-treated population.

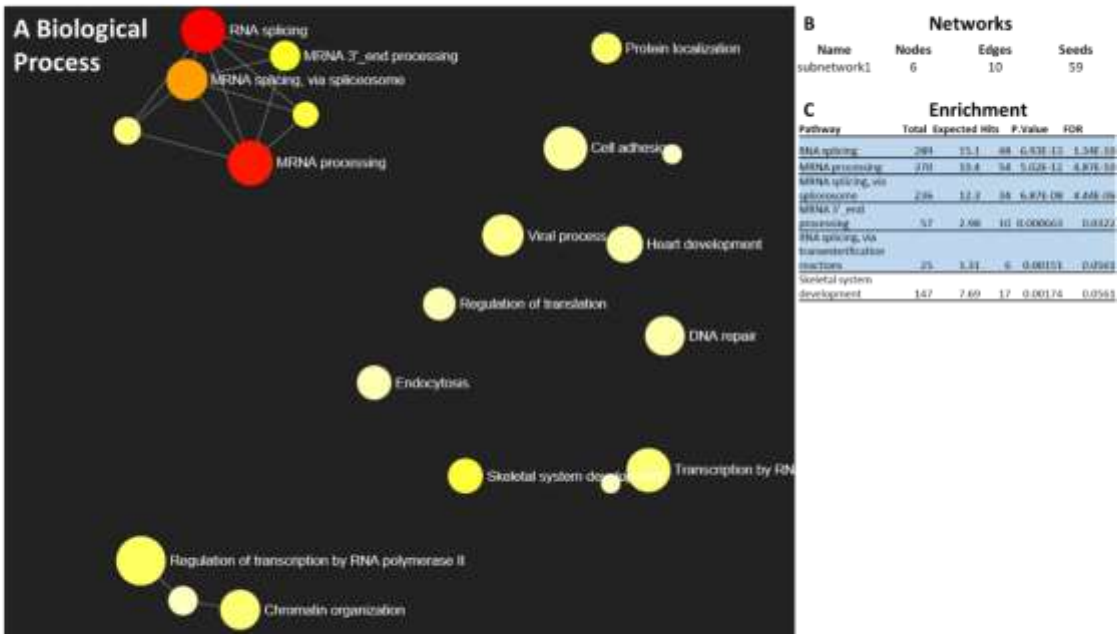

B **Networks**

Name	Nodes	Edges	Seeds
subnetwork1	6	10	59

C **Enrichment**

Pathway	Total	Expected	Hits	P.Value	FDR
RNA splicing	289	15.1	48	6.93E-13	1.34E-10
MRNA processing	370	19.4	54	5.02E-12	4.87E-10
MRNA splicing, via spliceosome	236	12.3	36	6.87E-08	4.44E-06
MRNA 3'_end processing	57	2.98	10	0.000063	0.0322
RNA splicing, via transesterification reactions	25	1.31	6	0.00151	0.0563
Skeletal system development	147	7.69	17	0.00174	0.0561

Figure S7. Enriched biological process network. (**A**) Network of enriched biological process terms in the differentially expressed gene list for the cluster 2 (4SC-202-treated) versus cluster 3 (control) comparison of the single-cell RNA-seq dataset. Color is related to the p-value from the over-representation analysis (ORA) with red being more significant and tan being less significant ($p < 0.05$). Size is directly related to the number of differentially expressed genes mapped to each term. Connections between terms indicate overlap in the gene list mapped to each term. (**B**) List of subnetworks in the biological process network. (**C**) Enriched terms and their significance (FDR < 0.05). Terms related to mRNA processing are highlighted blue.

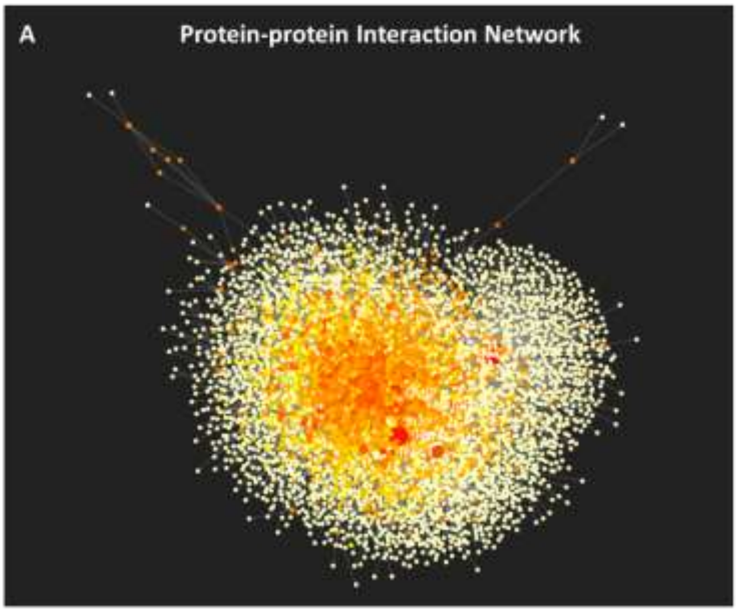

B **Networks**

Name	Nodes	Edges	Seeds
subnetwork1	6184	15043	765
subnetwork2	4	3	1
subnetwork3	3	2	1
subnetwork4	3	2	1
subnetwork5	3	2	1
subnetwork6	3	2	1

C **Enrichment**

Pathway	Total	Expected	Hits	P.Value	FDR
Viral process	448	163	329	1.29E-59	2.50E-57
Protein phosphorylation	627	228	374	3.43E-34	3.33E-32
Cell cycle	647	235	376	4.60E-31	2.97E-29
MRNA splicing, via spliceosome	[illegible]	85.7	[illegible]	[illegible]	[illegible]
DNA repair	[illegible]	[illegible]	[illegible]	1.97E-25	[illegible]
Negative regulation of apoptotic process	577	210	[illegible]	[illegible]	[illegible]
RNA splicing	289	105	186	1.22E-22	[illegible]
Chromatin organization	358	130	218	8.76E-22	3.12E-20
MRNA processing	320	[illegible]	210	[illegible]	[illegible]
Apoptotic process	[illegible]	[illegible]	[illegible]	[illegible]	[illegible]
Translation	315	114	190	1.72E-18	3.03E-17
Rhythmic process	124	45	90	1.72E-16	2.78E-15
Transcription by RNA polymerase II	[illegible]	[illegible]	324	[illegible]	[illegible]
Chromatin remodeling	91	33.1	68	[illegible]	[illegible]
Cell proliferation	386	140	208	7.01E-13	9.07E-12
Transcription, DNA_templated	217	78.8	110	2.50E-12	[illegible]
Endocytosis	184	66.8	113	2.99E-12	3.41E-11
Regulation of cell cycle	148	53.8	93	4.09E-11	4.39E-10
Protein transport	654	238	317	[illegible]	[illegible]
RNA metabolic process	47	17.1	37	3.31E-09	3.21E-08
Regulation of translation	139	50.5	84	5.53E-09	5.11E-08
Protein folding	157	57	92	9.45E-09	7.98E-08
RNA splicing, via transesterification reactions	25	[illegible]	[illegible]	[illegible]	[illegible]
Heart development	240	87.2	128	4.33E-08	3.50E-07
MRNA 3'_end processing	57	20.7	41	[illegible]	[illegible]
DNA replication	155	56.3	89	6.71E-08	4.63E-07
Cytoskeleton organization	170	61.8	94	3.06E-07	2.18E-06
Vesicle_mediated transport	274	99.5	140	3.14E-07	2.18E-06
Intracellular protein transport	354	128	173	[illegible]	[illegible]
Angiogenesis	252	91.5	128	1.50E-06	9.67E-06
Blood coagulation	193	70.1	101	3.48E-06	2.18E-05
Mitochondrion organization	90	32.7	54	3.92E-06	2.38E-05
Hemopoiesis	74	26.9	46	5.27E-06	3.10E-05
Regulation of catalytic activity	80	29.1	48	1.59E-05	7.61E-05
Nervous system development	516	187	232	2.34E-05	0.00013
Circadian rhythm	90	32.7	52	2.63E-05	0.000142
Chromosome segregation	83	30.1	48	5.13E-05	0.000269
DNA recombination	104	37.8	57	8.72E-05	0.000445
7_methylguanosine mRNA capping	34	12.4	23	2.00E-04	[illegible]
Regulation of DNA_binding transcription factor activity	24	8.72	17	0.000615	[illegible]
Protein targeting	44	16	27	0.000624	0.00255
Protein localization	76	27.6	41	0.00124	0.00566
Protein methylation	35	12.7	22	0.00125	0.00566
Cellular process	14	5.09	11	0.00157	0.00692
Cell differentiation	971	353	395	0.00193	0.00833
MRNA transcription	6	2.18	6	0.00229	[illegible]
Immune system process	515	187	217	0.00314	0.013
Cell_matrix adhesion	91	33.1	46	0.00371	0.015
Regulation of gene expression, epigenetic	16	5.81	11	0.0085	0.0334
Muscle contraction	111	40.3	53	0.00861	0.0334
Mesoderm development	37	13.4	21	0.00888	0.0344
Muscle organ development	104	37.8	50	0.00896	0.0344
Cellular protein modification process	110	40	52	0.0116	0.0426
Skeletal system development	147	53.4	67	0.0127	0.0456
Neuron_neuron synaptic transmission	9	3.27	7	0.014	0.0485
Chromatin assembly	9	3.27	7	0.014	0.0485
Regulation of binding	4	1.45	4	0.0174	0.0577
Nuclear transport	4	1.45	4	0.0174	0.0577
Receptor_mediated endocytosis	139	50.5	63	0.0175	0.0577
Response to toxic substance	98	35.6	46	0.0195	0.0632
Gluconeogenesis	44	16	23	0.0219	0.0698
Mammary gland development	35	12.7	19	0.0226	0.0706
Anterior/posterior axis specification	16	5.81	10	0.0298	0.0918
Ectoderm development	14	5.09	9	0.0312	0.0944

Figure S8. Cerebellum protein-protein interaction (ppi) network. (**A**) Network of protein-protein interactions initiated from differentially expressed genes (DEGs) in cluster 2 (4SC-202-treated) relative to cluster 3 (control) of the single-cell RNA-seq dataset. Only DEGs and proteins that directly interact with DEG seeds are included. The size of the node indicates the degree value and the color indicates the betweenness centrality values with red indicating higher centrality and tan indicating lower centrality. (**B**) List of subnetworks visualized in the ppi network. (**C**) Enriched terms and their significance (FDR < 0.05). Terms related to mRNA processing are highlighted blue, terms related to transcription regulation are highlighted green, and terms related to apoptosis are highlighted orange.

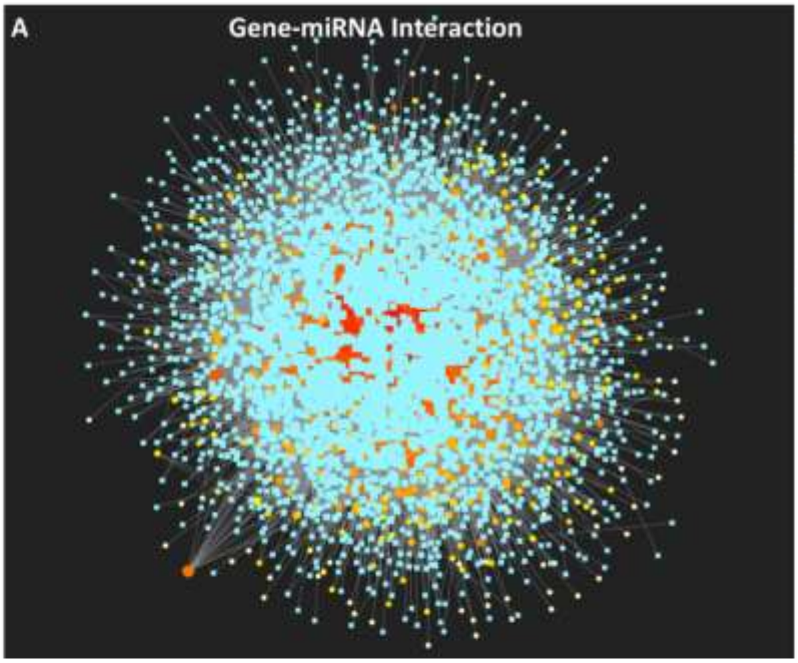

B Networks

Name	Nodes	Edges	Seeds
subnetwork1	3307	27212	841

C Enrichment

Pathway	Total	Expected	Hits	P.Value	FDR
RNA splicing	289	14.3	47	3.24E-13	6.28E-11
MRNA processing	370	18.3	53	1.75E-12	1.70E-10
MRNA splicing, via spliceosome	236	11.6	33	5.61E-08	[illegible]
MRNA 3'_end processing	57	2.81	10	0.00042	0.0204
Skeletal system development	147	7.26	17	0.000924	0.0358
Regulation of transcription by RNA polymerase II	1250	61.5	88	0.00179	0.0577

Figure S9. Gene-miRNA interaction network. (**A**) Network of gene-miRNA interactions seeded from differentially expressed genes in cluster 2 (4SC-202-treated) relative to cluster 3 (control) of the single-cell RNA-seq dataset (DEGs). Only miRNAs that are directly related to DEGs are included (blue squares). The size of DEGs (circles) indicate the degree value and the color indicates the betweenness centrality values with red indicating higher centrality and tan indicating lower centrality. (**B**) Subnetwork details for only subnetwork in the gene-miRNA interaction network. (**C**) Enriched terms and their significance (FDR < 0.05). Terms related to mRNA processing are highlighted blue and terms related to transcription regulation are highlighted green.

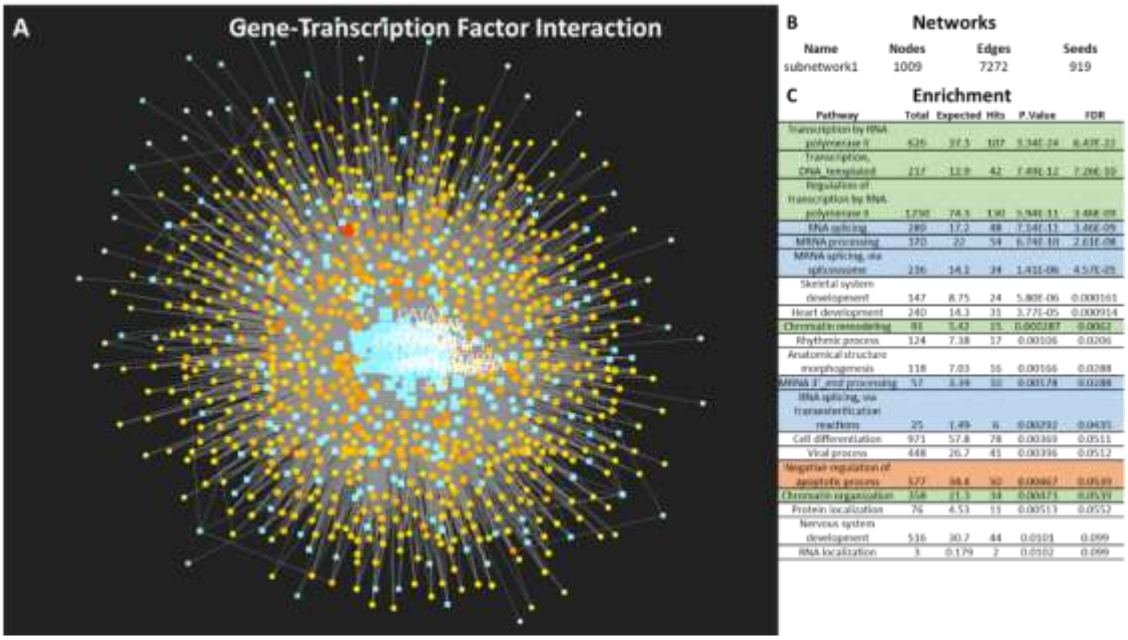

Figure S10. Gene-transcription factor interaction network. (**A**) Network of gene-transcription factor interactions seeded from differentially expressed genes (DEGs) in cluster 2 (4SC-202-treated) relative to cluster 3 (control) of the single cell RNA-seq dataset. Only DEGs (color circles) and transcription factors (blue squares) that directly interact with them are included in the network. Circle size is determined by the degree value and circle color indicates the betweenness centrality values with red indicating higher centrality and tan lower centrality. (**B**) Subnetwork details for only subnetwork in the gene-transcription factor interaction network. (**C**) Enriched terms and their significance (FDR < 0.05). Terms related to mRNA processing are highlighted blue, terms related to transcription regulation are highlighted green, and terms related to apoptosis are highlighted orange.

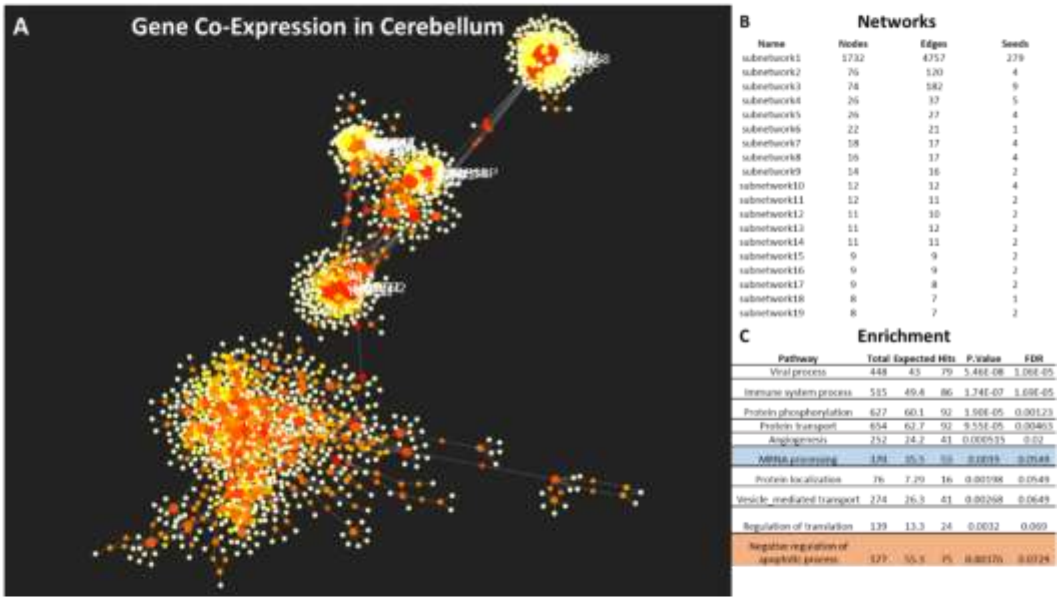

Figure S11. Cerebellum gene co-expression network. (**A**) Network of co-expressed genes seeded Figure 2. (4SC-202-treated) relative to cluster 3 (control) in the single-cell RNA-seq dataset. Node size is determined by degree value and node color by betweenness centrality. Red nodes are more central than are lighter colored nodes. (**B**) Details of subnetworks in the gene co-expression network. (**C**) Enriched terms and their significance (FDR < 0.05). Terms related to mRNA processing are highlighted blue and terms related to apoptosis are highlighted orange.

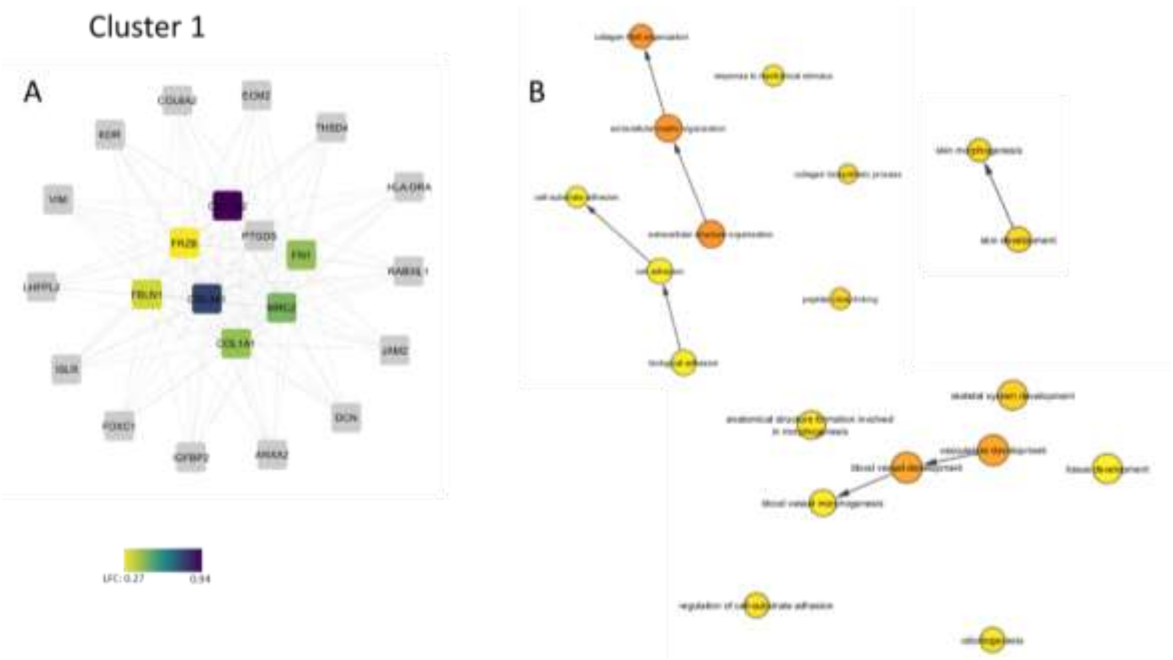

Figure S12. Densely connected subregion (cluster) 1 and biological process enrichment from integrated systems biology analysis (**A)** Network of bioelements and their interactions. The MCODE score for this cluster is 11.905 based on the size and density of the subgraph. Lines are unweighted and indicate interactions in the integrated systems biology network (Figure 7). Color is based on the log fold change of the gene expression between cluster 2 and cluster 3 of the single cell dataset. Grey squares indicate genes that were not differentially expressed. (**B**) Overrepresented GO terms for the densely connected subgraph ($p < 0.05$) and their ontological hierarchy (arrows). Coloring of GO nodes indicates significance where red is high and yellow is low and the size of the circles indicate how many differentially expressed genes were mapped to each term.

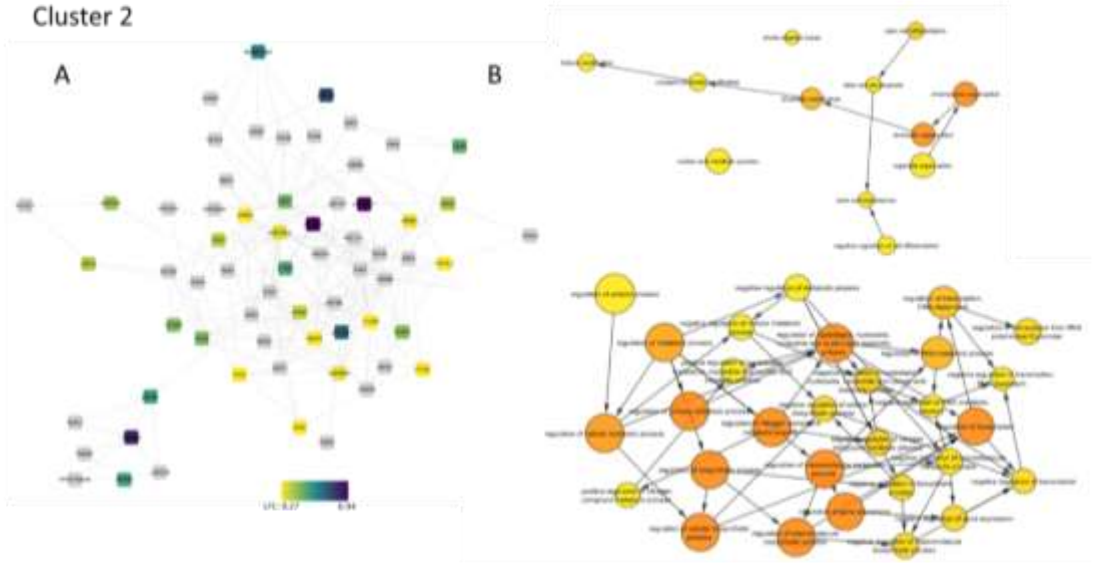

Figure S13. Densely connected subregion (cluster) 2 and biological process enrichment from integrated systems biology analysis (**A**) Network of bioelements and their interactions. The MCODE score for this cluster is 6.469 based on the size and density of the subgraph. Lines are unweighted and indicate interactions in the integrated systems biology network (Figure 7). Color is based on the log fold change of the gene expression between cluster 2 and cluster 3 of the single cell dataset. Grey squares indicate genes that were not differentially expressed. (**B**) Overrepresented GO terms for the densely connected subgraph ($p < 0.05$) and their ontological hierarchy (arrows). Coloring of GO nodes indicates significance where red is high and yellow is low and the size of the circles indicate how many differentially expressed genes were mapped to each term.

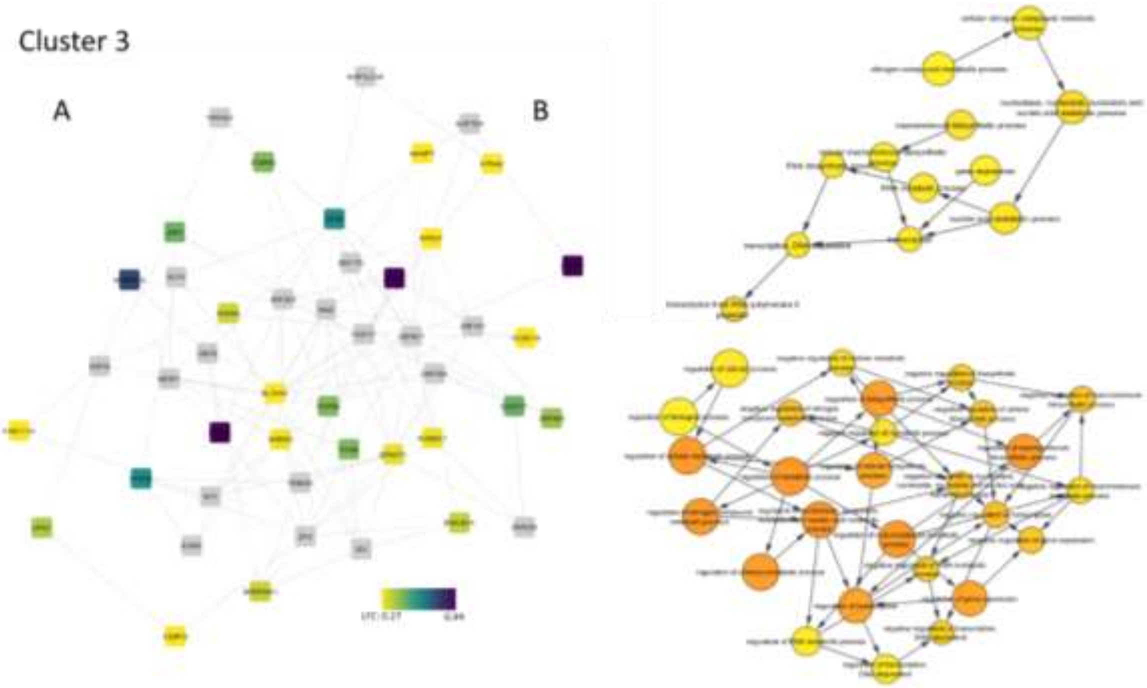

Figure S14. Densely connected subregion (cluster) 3 and biological process enrichment from integrated systems biology analysis (**A**) Network of bioelements and their interactions. The MCODE score for this cluster is 5.689 based on the size and density of the subgraph. Lines are unweighted and indicate interactions in the integrated systems biology network (Figure 7). Color is based on the log fold change of the gene expression between cluster 2 and cluster 3 of the single cell dataset. Grey squares indicate genes that were not differentially expressed. (**B**) Overrepresented GO terms for the densely connected subgraph ($p < 0.05$) and their ontological hierarchy (arrows). Coloring of GO nodes indicates significance where red is high and yellow is low and the size of the circles indicate how many differentially expressed genes were mapped to each term.

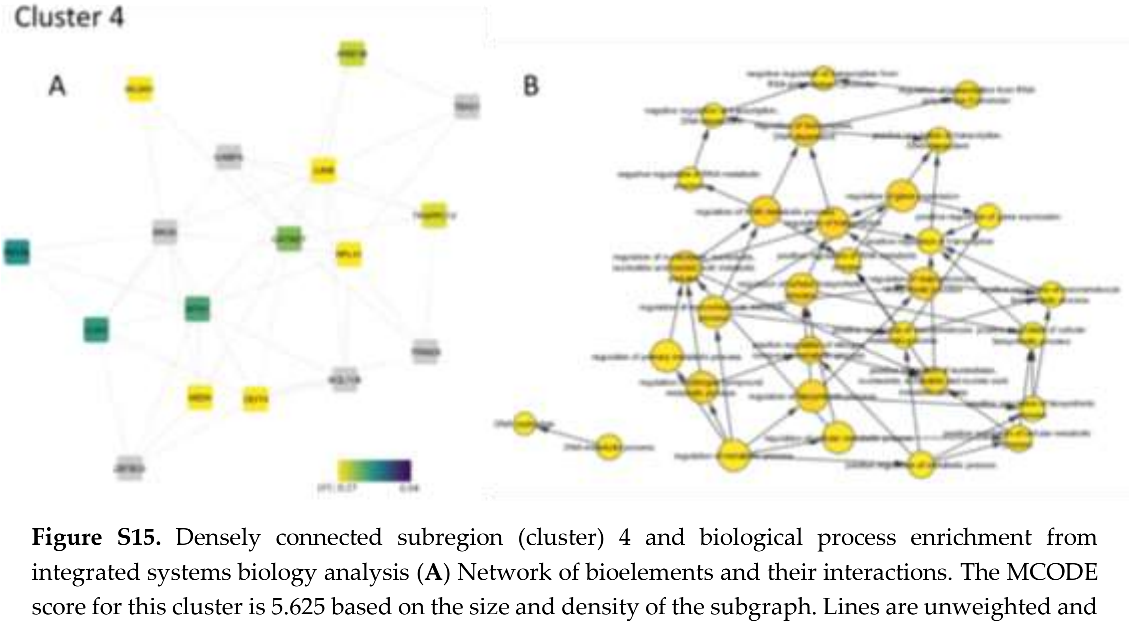

Figure S15. Densely connected subregion (cluster) 4 and biological process enrichment from integrated systems biology analysis (**A**) Network of bioelements and their interactions. The MCODE score for this cluster is 5.625 based on the size and density of the subgraph. Lines are unweighted and indicate interactions in the integrated systems biology network (Figure 7). Color is based on the log fold change of the gene expression between cluster 2 and cluster 3 of the single cell dataset. Grey squares indicate genes that were not differentially expressed. (**B**) Overrepresented GO terms for the densely connected subgraph ($p < 0.05$) and their ontological hierarchy (arrows). Coloring of GO nodes indicates significance where red is high and yellow is low and the size of the circles indicate how many differentially expressed genes were mapped to each term.

Cluster 5

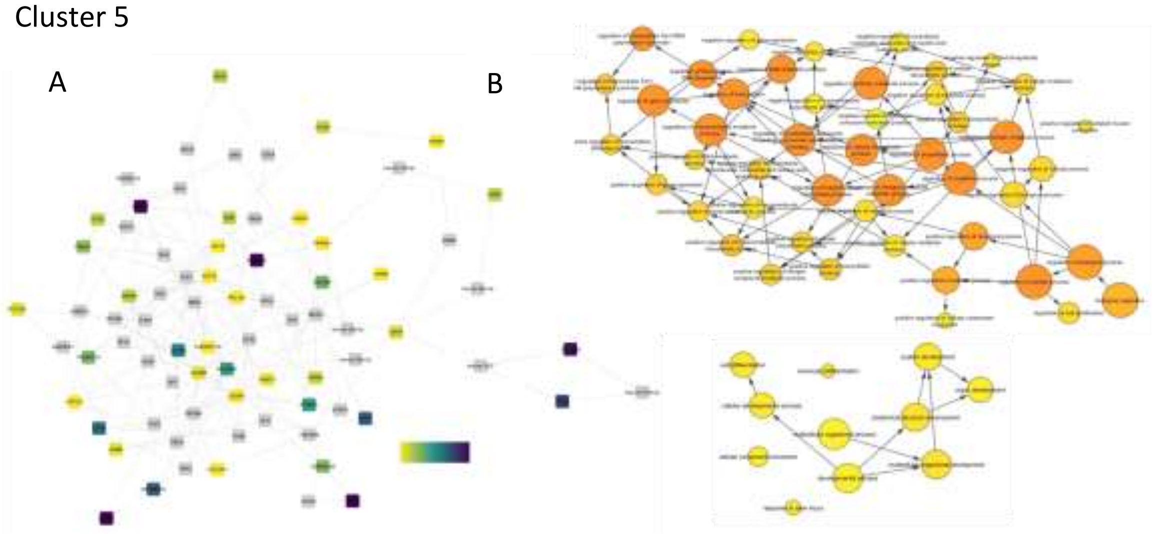

Figure S16. Densely connected subregion (cluster) 5 and biological process enrichment from integrated systems biology analysis (**A**) Network of bioelements and their interactions. The MCODE score for this cluster is 4.947 based on the size and density of the subgraph. Lines are unweighted and indicate interactions in the integrated systems biology network (Figure 7). Color is based on the log fold change of the gene expression between cluster 2 and cluster 3 of the single cell dataset. Grey squares indicate genes that were not differentially expressed. (**B**) Overrepresented GO terms for the densely connected subgraph ($p < 0.05$) and their ontological hierarchy (arrows). Coloring of GO nodes indicates significance where red is high and yellow is low and the size of the circles indicate how many differentially expressed genes were mapped to each term.

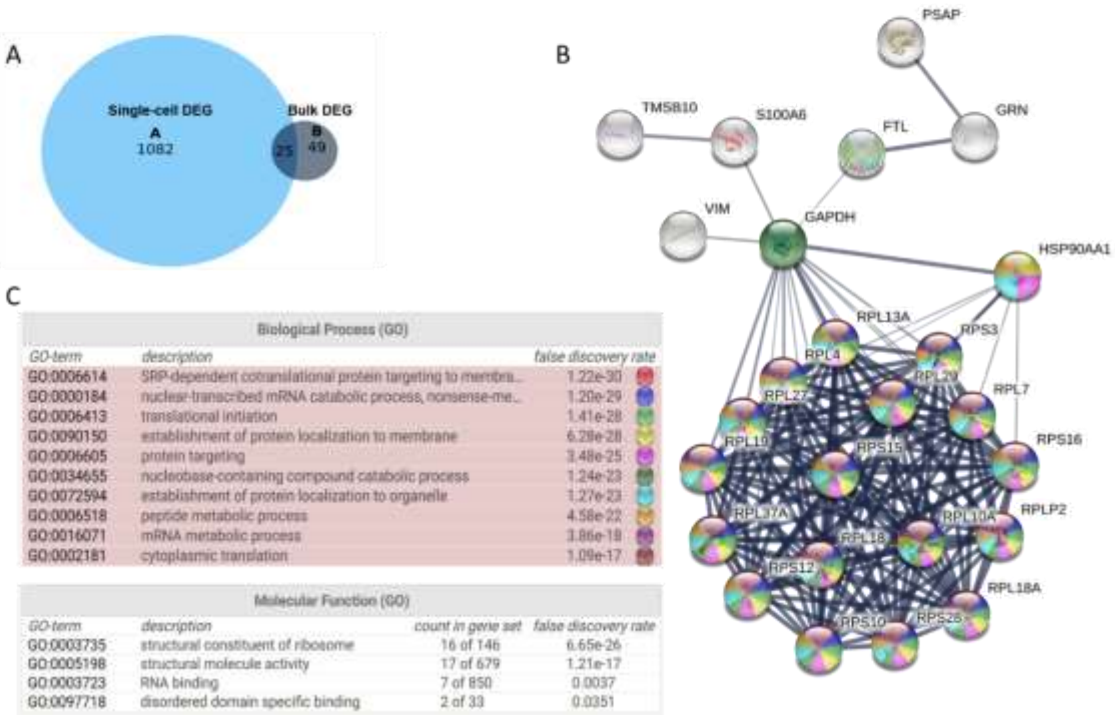

Biological Process (GO)		
GO-term	description	false discovery rate
GO:0006614	SRP-dependent cotranslational protein targeting to membra...	1.22e-30
GO:0000184	nuclear-transcribed mRNA catabolic process, nonsense-me...	1.20e-29
GO:0006413	translational initiation	1.41e-28
GO:0090150	establishment of protein localization to membrane	6.28e-28
GO:0006605	protein targeting	3.48e-25
GO:0034655	nucleobase-containing compound catabolic process	1.24e-23
GO:0072594	establishment of protein localization to organelle	1.27e-23
GO:0006518	peptide metabolic process	4.58e-22
GO:0016071	mRNA metabolic process	3.86e-18
GO:0002181	cytoplasmic translation	1.09e-17

Molecular Function (GO)			
GO-term	description	count in gene set	false discovery rate
GO:0003735	structural constituent of ribosome	16 of 146	6.65e-26
GO:0005198	structural molecule activity	17 of 679	1.21e-17
GO:0003723	RNA binding	7 of 850	0.0037
GO:0097718	disordered domain specific binding	2 of 33	0.0351

Figure S17. Genes differentially expressed between 4SC-202-treated and control spheroids in both single-cell and bulk RNA-sequencing datasets are enriched for ribosome-related GO terms. (**A**) Venn diagram of differentially expressed genes for bulk and single cell sequencing results (FDR < 0.05, ∣LFC∣ > 0.25, LFC restriction imposed for single cell dataset only). (**B**) STRING 11.0 diagram of 25 genes differentially expressed in both the single cell RNA-sequencing and the bulk RNA-sequencing datasets [2]. Protein nodes are colored by the enriched GO biological process terms they are involved in and edge thickness indicates the strength of the evidence of the interaction between proteins. (**C**) Enriched GO biological process terms and GO molecular function terms. Color of biological process terms corresponds to color of protein nodes. FDR < 0.05. Only the first 10 terms are shown of the enriched GO biological process terms. 16 of the 25 terms are mapped to structural constituent of ribosome, and enriched biological process terms include terms related to translation, translocation of proteins, and mRNA catabolic processes.

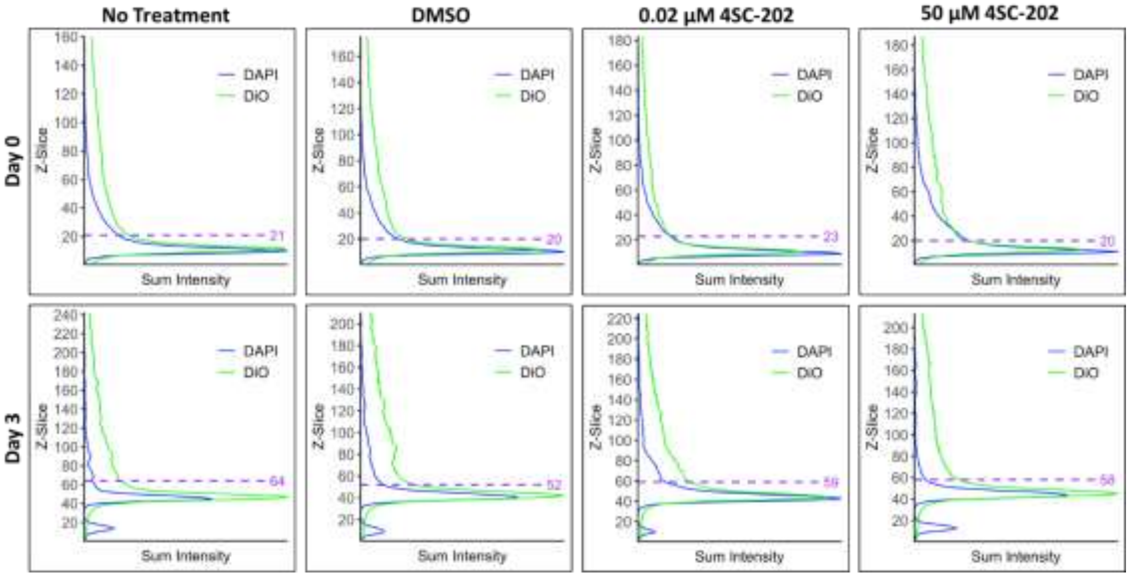

Figure S18. Cropping location relative to the total intensity along the z-axis. Each slice's pixel intensities were summed into two values per slice: one for sum DAPI intensity, and one for sum DiO intensity. The blue line indicates DAPI channel slice intensities, and the green line indicates DiO channel slice intensities. Dashed purple lines indicate crop points along the z-stacks. Slice numbers are indicated to the right of each dashed line. For image pre-processing, slices less than the indicated value were removed.

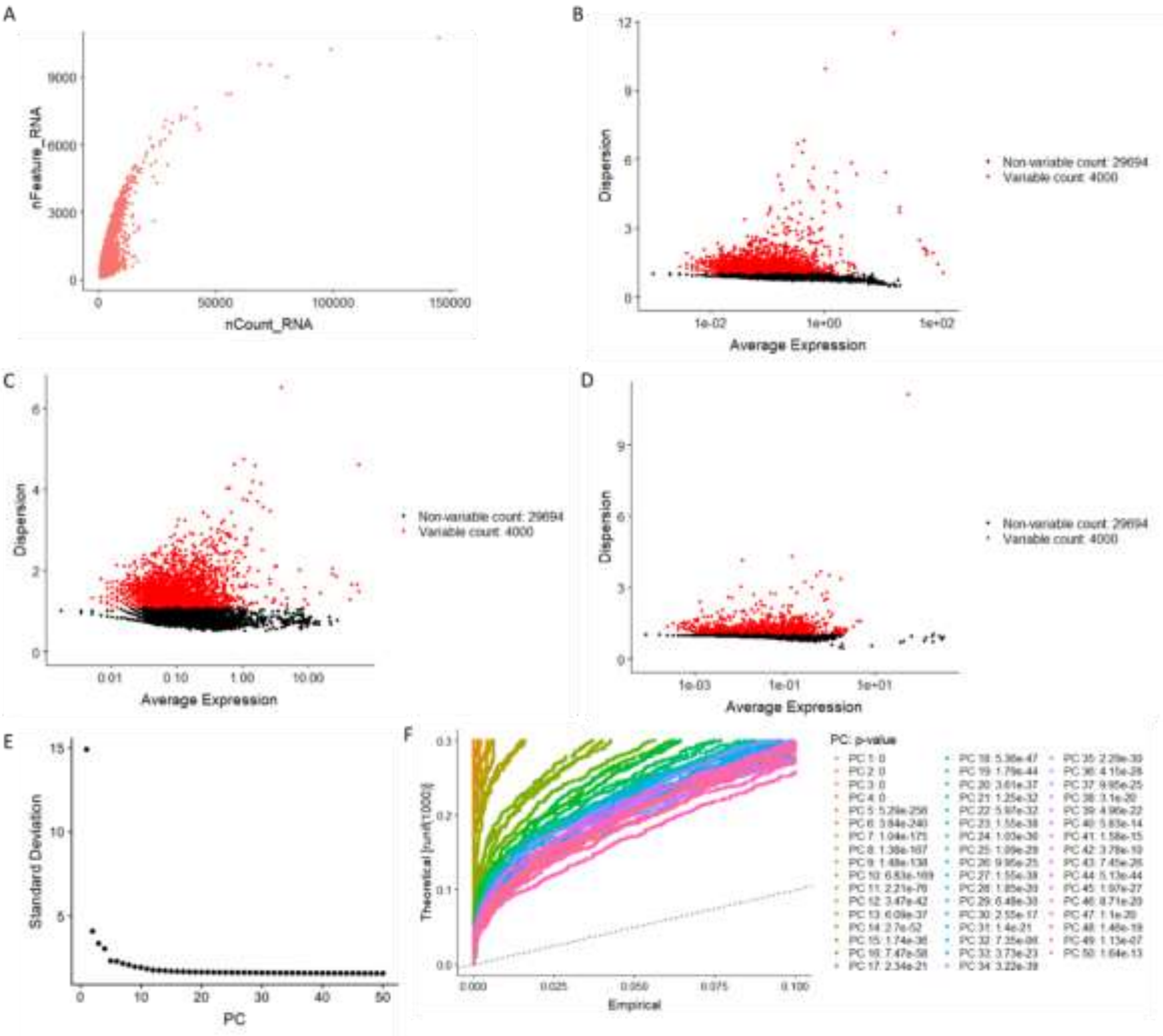

Figure S19. Parameter selection plots for single cell analysis in Seurat [3]. (**A**) Number of features versus number of RNA molecule plot for outlier identification. Cells with less than 40,000 counts of RNA were kept for analysis. (**B**–**D**) Variable feature plots of 4000 retained features for (**B**) small control spheroid, (**C**) control spheroid, and (**D**) 4SC-202-treated spheroid. (**E**) Elbow plot showing the standard deviations of the first 50 principle components (PCs). (**F**) Jack Straw Analysis and p values for the first 50 PCs. Jack Straw Q-Q plot shows distribution of p values for each PC relative to a theoretical uniform distribution (dashed line). P values are based on a proportion test of the number of significant p values in the PC compared to the theoretical uniform distribution.

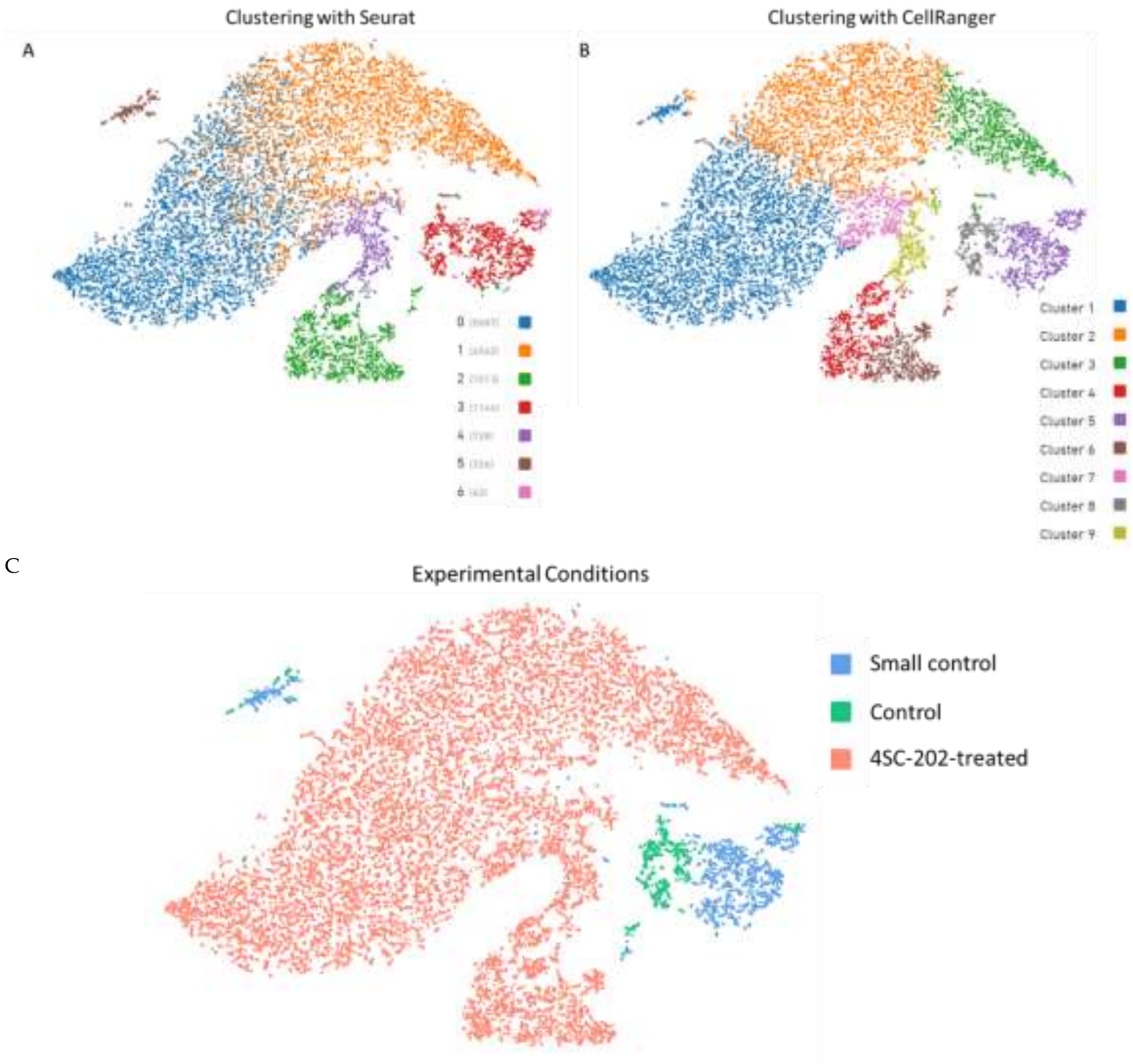

Figure S20. Seurat graph-based clusters correlate with clusters identified using the CellRanger pipeline. (**A**) 10× Genomics Loupe Cell Browser TSNE plot of scRNA-seq data with colors indicating the clusters calculated using the Seurat algorithm (**B**) Loupe Cell Browser TSNE plot of scRNA-seq data with colors indicating the clusters identified using the CellRanger pipeline. While Seurat and CellRanger clusters identify different numbers of clusters, the CellRanger clusters tend to be sub-clusters of the Seurat clusters. One notable exception is Cluster 6, which appears in the Seurat clustering but not in the CellRanger clustering. Cluster 6 does occupy a distinct area in the TSNE plot calculated by the CellRanger pipeline. (**C**) Loupe Cell Browser TSNE plot of experimental conditions.

References:

1. Messerli, S.M.; Hoffman, M.M.; Gnimpieba, E.Z.; Kohlhof, H.; Bhardwaj, R.D. 4SC-202 as a potential treatment for the pediatric brain tumor medulloblastoma. *Brain Sci.* **2017**, doi:10.3390/brainsci7110147
2. Szklarczyk, D.; Gable, A.L.; Lyon, D.; Junge, A.; Wyder, S.; Huerta-Cepas, J.; Simonovic, M.; Doncheva, N.T.; Morris, J.H.; Bork, P.; et al. STRING v11: Protein-protein association networks with increased coverage, supporting functional discovery in genome-wide experimental datasets. *Nucleic Acids Res.* **2019**, *47*, D607–D613.
3. Stuart, T.; Butler, A.; Hoffman, P.; Hafemeister, C.; Papalexi, E.; Mauck, W.M.; Hao, Y.; Stoeckius, M.; Smibert, P.; Satija, R. Comprehensive Integration of Single-Cell Data. *Cell* **2019**, *177*, 1888–1902.e21.
4. Butler, A.; Hoffman, P.; Smibert, P.; Papalexi, E.; Satija, R. Integrating single-cell transcriptomic data across different conditions, technologies, and species. *Nat. Biotechnol.* **2018**, *36*, 411–420.
5. Gu, Z.; Eils, R.; Schlesner, M. Complex heatmaps reveal patterns and correlations in multidimensional genomic data. *Bioinformatics* **2016**, *32*, 2847–2849.
6. Zhou, G.; Soufan, O.; Ewald, J.; Hancock, R.E.W.; Basu, N.; Xia, J. NetworkAnalyst 3.0: a visual analytics platform for comprehensive gene expression profiling and meta-analysis. *Nucleic Acids Res.* **2019**, *47*, W234–W241.
